# Profiler: an open web platform for multi-omics analysis

**DOI:** 10.1093/bioinformatics/btaf644

**Published:** 2025-12-01

**Authors:** Yanis Zirem, Léa Ledoux, Isabelle Fournier, Michel Salzet

**Affiliations:** Univ. Lille, Inserm, CHU Lille, U1192, Protéomique Réponse Inflammatoire Spectrométrie de Masse, PRISM, Lille F-59000, France; Univ. Lille, Inserm, CHU Lille, U1192, Protéomique Réponse Inflammatoire Spectrométrie de Masse, PRISM, Lille F-59000, France; Univ. Lille, Inserm, CHU Lille, U1192, Protéomique Réponse Inflammatoire Spectrométrie de Masse, PRISM, Lille F-59000, France; Institut Universitaire de France, ministère de l’Enseignement supérieur, de la Recherche et de l’Innovation, Paris Cedex 05 75231, France; Univ. Lille, Inserm, CHU Lille, U1192, Protéomique Réponse Inflammatoire Spectrométrie de Masse, PRISM, Lille F-59000, France; Institut Universitaire de France, ministère de l’Enseignement supérieur, de la Recherche et de l’Innovation, Paris Cedex 05 75231, France

## Abstract

**Motivation:**

High-throughput multi-omics technologies produce increasingly large and heterogeneous datasets that are difficult to analyze without advanced computational expertise. Existing bioinformatics tools are often fragmented or limited to specific omics types, hindering reproducibility and accessibility. There is a critical need for an integrated, user-friendly, and scalable platform capable of supporting multi-omics analyses across different data modalities.

**Results:**

We present Profiler, an open-source, modular platform that unifies data import, quality control, preprocessing, statistical testing, machine and deep learning, biomarker discovery, pathway and drug–target enrichment, and survival modeling within a single reproducible environment. Built in Python with Streamlit, Profiler is available as both a web-based platform deployed on high-performance computing and a desktop version for local execution, enabling flexible usage across computational infrastructures. Profiler supports diverse omics modalities, including proteomics, transcriptomics, lipidomics, and electroencephalogram data. Through applications to glioblastoma proteomic, pancancer, and multi-omics datasets, Profiler reproduced known molecular subtypes, revealed potential therapeutic targets, and generated fully traceable analysis reports within minutes. By integrating advanced analytics behind an intuitive interface, Profiler democratizes multi-omics analysis and provides a robust, scalable foundation for systems biology and precision medicine research.

**Availability and implementation:**

Profiler is open-source and freely available via its web platform (https://prism-profiler.univ-lille.fr) and GitHub (web version: https://github.com/yanisZirem/Profiler_v1_requests_datatests, desktop version: https://github.com/yanisZirem/prism-profiler), and archived on Zenodo (DOI: https://doi.org/10.5281/zenodo.17478158).

## 1 Introduction

The advent of high-throughput technologies, such as next-generation sequencing (NGS), mass spectrometry (MS) and microarrays, has revolutionized biomedical research. These platforms generate large-scale, multi-dimensional datasets, collectively referred to as omics data, encompassing genomics, transcriptomics, proteomics, and metabolomics. Such datasets hold immense potential for elucidating biological mechanisms, discovering disease biomarkers, and identifying novel therapeutic targets. However, the complexity, heterogeneity, and volume of omics data introduce substantial computational and analytical challenges (Hasin *et al.* 2017).

Traditional omics data analysis typically requires specialized expertise in bioinformatics, statistics, and programming, placing it beyond the reach of many experimental biologists and clinicians. Furthermore, many existing tools are limited in scope, tailored to specific omics types or single-step analyses and are often confined to command-line environments, which hinder accessibility, interoperability and reproducibility. Researchers are frequently compelled to navigate fragmented workflows across multiple software packages, leading to inefficiencies, steep learning curves, and reproducibility concerns (Perez‐Riverol *et al.* 2015, Mangul *et al.* 2019).

In response to these limitations, there is a growing demand for integrated, user-friendly, and visually intuitive platforms that combine analytical robustness with accessibility. Solutions such as Galaxy (Afgan *et al.* 2018), MetaboAnalyst (Pang *et al.* 2021), and Perseus (Tyanova *et al.* 2016b) have made important strides in addressing specific areas of omics analysis. However, few platforms offer a truly comprehensive, end-to-end solution that covers multiple omics modalities, incorporates advanced machine learning (ML) and deep learning (DL) methods, and enables interactive data visualization and interpretation within a unified environment.

To address these critical gaps, we introduce Profiler, a modular, web-based application designed to democratize omics data analysis. Developed in Python using the Streamlit framework, Profiler provides a seamless and integrated pipeline covering key stages of analysis: data import and conversion, preprocessing (including cleaning, normalization, imputation, batch effect correction), visualization, statistical testing, ML, DL, biomarker discovery, pathway enrichment analysis, and survival analysis. The platform is built for scalability, modularity, and extensibility, allowing it to evolve with emerging research needs and analytical innovations.

Notably, Profiler is engineered to serve both novice and expert users. It offers guided, workflow-oriented interfaces for users with limited computational experience, while its flexible architecture supports customization and advanced analytical workflows for experienced users. Profiler’s compatibility with a wide range of data formats, combined with efficient backend processing, ensures robust performance even with high-dimensional datasets.

By lowering the technical barriers to entry, Profiler aims to provide the scientific community with an accessible, transparent, and comprehensive analytical ecosystem, one that promotes reproducibility, accelerates discovery, and empowers data-driven decision-making in modern life sciences research.

## 2 Materials and methods

The dataset used in this article to demonstrate the utility of Profiler originates from the studies by Duhamel *et al.* (2022) and Lagache *et al.* (2025). While the data were initially collected in 2022, they were reanalyzed in the 2025 study using a more appropriate and advanced data analysis pipelines.

### 2.1 Cohort

Tumors from 50 patients were included in the study. Patients with newly diagnosed glioblastoma were prospectively enrolled between September 2014 and November 2018 at Lille University Hospital, France (NCT02473484). All patients gave written informed consent before enrollment. These 50 tumors were used for omics MALDI-MSI and proteomics analysis. Tumors samples were processed within 2 h after sample extraction in the surgery room to limit the risk of degradation of proteins.

### 2.2 Spatially resolved proteomics extraction

The different clusters identified by the segmentation process [detailed explanation in Lagache *et al.* (2025)] were submitted to spatially resolved proteomics. A localized digestion was carried out by deposing a trypsin solution (40 μg/ml in NH_4_HCO_3_ 50 mM), on a region of 500 μm^2^ of tissue (4 × 4 droplets of 200 μm in diameter), using CHIP-1000. The deposition method comprises approximately 1205 cycles per digestion spot, i.e. 3 h of deposition, with a drop volume of 150 pl. Finally, each spot was digested with 0.112 μg of trypsin. Following the micro-digestion, each spot was extracted by liquid microjunction using the TriVersa Nanomate device, with LESA (Liquid Extraction and Surface Analysis) parameters (Quanico *et al.* 2013). The tryptic peptides were extracted by performing two consecutive extraction cycles for three different solvents mixtures [TFA 0.1%; ACN/0.1% TFA (8:2, v/v); and MeOH/0.1% TFA (7:3, v/v)] for a total of six extractions. For each cycle, 2 μl of solvent was drawn into the tip of the pipette, of which 0.8 pl was brought into contact with the surface. Fifteen back and forth movements were performed to extract the peptides before collecting the solution in a recovery tube. All extracts were pulled in one tube and 50 μl of ACN were finally added before drying the samples in a SpeedVac. The samples were then stored at −20°C prior to nLC–MS/MS analysis.

### 2.3 nLC–MS/MS bottom-up analysis

Prior to MS analysis, the reconstituted samples were desalted using C18 Ziptip (Millipore, Saint-Quentin-en-Yvelines, France), eluted with 80% ACN and vacuum-dried. The dried samples were resuspended in 0.1% FA aqueous/ACN (98:2, v/v). Peptides separation was performed by reverse phase chromatography, using a NanoAcquity UPLC system (Waters) coupled to a Q-Exactive Orbitrap mass spectrometer (Thermo Scientific) via a nanoelectrospray source. A pre-concentration column (nanoAcquity Symmetry C18, 5 µm, 180 µm × 20 mm) and an analytical column (nanoAcquity BEH C18, 1.7 µm, 75 µm × 250 mm) were used. A 2 h linear gradient of acetonitrile in 0.1% formic acid (5%–35%) was applied, at the flow rate of 300 nl/min. For MS and MS/MS Acquisition (Xcalibur 4.1 and Exactive Series 2.9), a data-dependent mode was defined to analyze the 10 most intense ions of MS analysis (Top 10). The MS analysis was performed with an *m/z* mass range between 300 and 1600, a resolution of 70 000 FWHM, an AGC of 3e^6^ ions and a maximum injection time of 120 ms. The MS/MS analysis was performed with an *m/z* mass range between 200 and 2000, an AGC of 5e^4^ ions, a maximum injection time of 60 ms, and the resolution was set at 17 500 FWHM. To avoid any batch effect during the analysis, the extractions were chosen at random to create analysis sequences.

### 2.4 Data analysis prior to the use of Profiler (2022 study)

All MS data were searched with MaxQuant software (Tyanova *et al.* 2016a) (Version 1.5.3.30) using Andromeda search engine against the complete proteome for Homo sapiens (UniProt, release July 2018, 20 412 entries). Trypsin was selected as enzyme and two missed cleavages were allowed, with N-terminal acetylation and methionine oxidation as variable modifications. The mass accuracies were set to 6 and 20 ppm, respectively, for MS and MS/MS spectra. False discovery rate (FDR) at the peptide spectrum matches (PSM) and protein levels was estimated using a decoy version of the previously defined databases (reverse construction, Homo sapiens, UniProt, release July 2018) and set to 1%. A minimum of two peptides with at least one unique is necessary to complete the identification of a protein. The MaxLFQ algorithm was used to perform label-free quantification (LFQ) of the proteins.

### 2.5 Data analysis prior to the use of Profiler (2025 study)

The aim of the 2025 study (Lagache *et al.* 2025) was to advance the concept of dry proteomics. To this end, lipidomic and proteomic MALDI-MSI analyses were performed on 13 glioblastoma tissue samples. Common molecular clusters were identified and correlated with micro-proteomic data previously obtained in the 2022 study (Duhamel *et al.* 2022).

We developed a dedicated pipeline for the segmentation of MS imaging (MSI) data to assess the number and spatial distribution of tumor clones, both within and across patients. A t-SNE analysis based on lipidomic imaging data revealed a clear separation into two distinct groups. This clustering pattern was consistently recapitulated in the heatmap derived from micro-proteomic data, further supporting the robustness of the classification. As a result, the samples were stratified into two molecular subgroups, referred to as group A and group B. Comparison of clinical outcomes and differential analysis showed that group A was associated with significantly longer overall survival (greater than 32 months) and tumor aggressiveness, invasion, and therapeutic resistance, while group B was linked to a poorer prognosis (survival less than 30 months) and less aggressiveness, necrosis, and potential therapeutic targets. To automate the prediction of patient outcomes, we developed a dry-lab proteomic analysis pipeline. This pipeline enabled the extraction of spatially resolved MSI clusters, which were subsequently analyzed using trained ML models. From a single pixel or an MSI-derived cluster, the models could predict the identity of the corresponding tumor clone, its associated protein expression profile, its classification into group A or B, and ultimately the patient’s prognostic category. The two groups, A and B, and their association with patient survival were validated in an independent cohort of 37 patients. Accordingly, the dataset analyzed in this study comprises the 50 patients classified into groups A and B.

**Figure 1.**
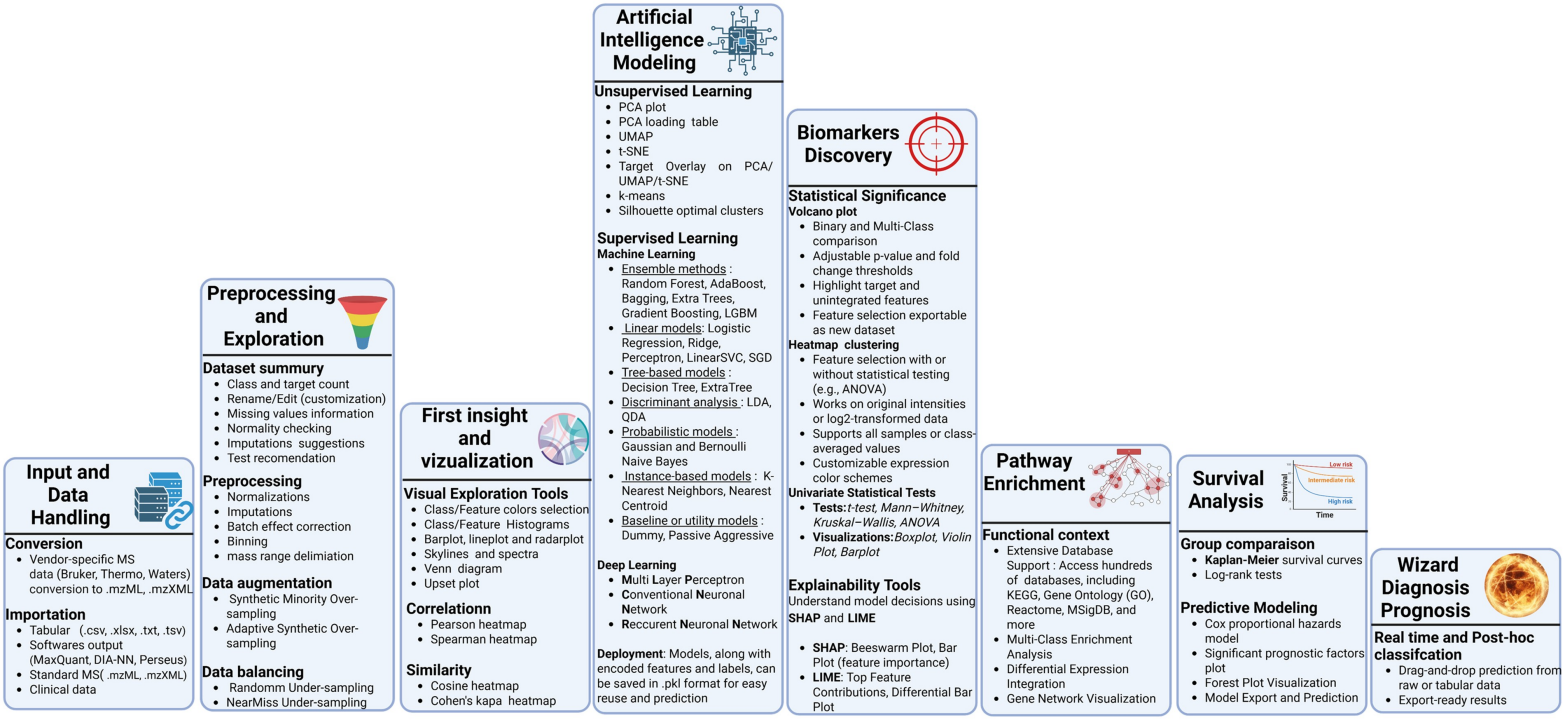
End-to-end profiler analysis pipelines. Modular architecture with streamlined flow through eight interconnected components. Created in https://BioRender.com.

Although the study included 50 patients, analyses were performed at the tumor clone level. Multiple clones were characterized per patient and grouped into two biologically defined classes, aggressive and non-aggressive, based on survival correlation and pathway analysis. Each clone was treated as an independent sample, never split across folds, ensuring biologically consistent training and validation (see Fig. 1, available as supplementary data at *Bioinformatics* online).

### 2.6 Profiler privacy and data protection statement

Profiler operates as a secure web-based application. All data uploaded through the interface are processed locally within the user session for analysis and visualization purposes only. No patient, lipidomic, proteomic, or genomic data are stored, transmitted, or retained on external servers after the session ends. Uploaded datasets remain entirely under the user’s control and are not accessible to any third party. Profiler does not store or share uploaded data, aligning with good data governance and privacy practices.

### 2.7 Scalability and system resources

Profiler is designed with scalability and robust system resources in mind, ensuring optimal performance and reliability for high-demand analytical tasks. The platform runs on the Mésocentre de Calcul de Lille (https://hpc.univ-lille.fr), leveraging an infrastructure that includes 246 GB of RAM, 8 GB of swap memory and multiple vCPUs and vGPUs. This setup, operating on a Linux server and utilizing OpenStack cloud technology, provides ample computational power to handle complex analyses efficiently. Additionally, Profiler benefits from expandable GPU and vCPU pools, allowing for dynamic scaling of resources based on user demand. The system is actively monitored to ensure it meets the analytical needs of its users. If user demand increases, as tracked via system telemetry, Profiler’s compute resources (CPU, GPU, RAM) can be scaled in collaboration with high-performance computing (HPC) administrators. This proactive approach ensures that the platform remains responsive and capable of handling increased loads without compromising performance. Furthermore, plans are in place to augment the system’s capacity if there is a surge in demand from the user community, ensuring that Profiler continues to deliver high-quality, timely results even under heavy usage.

The technological stack supporting Profiler’s backend, frontend, and cloud infrastructure is detailed in Table 1, which outlines the key libraries and tools integrated into the platform to enable efficient data processing, modeling, visualization, and deployment.

**Table 1.** Overview of technologies and libraries used in Profiler platform.

	Technology/library	Description/role
Backend	Python	Main programming language, integrates all modules and orchestrates workflow execution
	Pandas	Data manipulation and preprocessing for tabular and omics data
	Numpy	Efficient numerical operations and array manipulation
	pyopenMS	Mass spectrometry file parsing
	Msconvert (ProteoWizard)	Raw MS data conversion to open formats
	Openpyxl	Excel file handling
	Scikit-learn	Machine learning models
	Tensorflow/Keras	Deep learning model design and training (MLPs, CNNs, RNNs)
	Lifelines	Survival modeling and stratification
	Imbalanced-learn	Class balancing (e.g. SMOTE, ADASYN, under-sampling)
	Pycombat	Batch effect correction
	SHAP, Eli5	Model explainability
	Scipy.stats, statsmodels	Parametric and non-parametric statistical tests (e.g. *t*-test, ANOVA, Kruskal–Wallis, Mann–Whitney)
	Joblib/pickle	Model serialization and persistence (saving/loading ML pipelines and objects)
	GSEApy	Gene set enrichment analysis
	NetworkX	Construction and analysis of biological networks and pathway graphs
Frontend	Streamlit	Web-based user interface
	HTML.CSS	Custom layout and styling of the interface components
	Plotly, Matplotlib, Seaborn	Interactive and static visualizations (e.g. spectra, volcano plots, radar charts)
HPC and cloud	Linux (ubuntu)	Operating system for server and local environments
	Open stack	Cloud infrastructure management for resource provisioning
	Systemd	Service orchestration and daemon management
	Nginx	Reverse proxy server for deployment, load balancing, and API exposure
	Docker	Containerization for reproducibility, environment control, and deployment

## 3 Results

Profiler’s primary goal is to bridge the gap between raw omics data and actionable biological insights by leveraging a custom pipeline combining state-of-the-art libraries, original modules, and HPC. Figure 1 illustrates the eight interconnected components of this software [detailed in the User’s Manual downloadable via GitHub (https://github.com/yanisZirem/prism-profiler) or directly from the software’s home page (https://prism-profiler.univ-lille.fr)]. To improve accessibility for readers from diverse scientific backgrounds, Table 1, available as supplementary data at *Bioinformatics* online, provides a glossary defining technical key terms related to multi-omics analysis, ML, and DL.

To demonstrate how Profiler operates and the types of results it can generate, the proteomic dataset processed with MaxQuant will be used as main running example throughout the workflow. Additionally, lipidomic data acquired using the SpiderMass technology, such as those published by (Zirem *et al.* 2024a) will be used for real-time wizard module not useful for proteomic dataset. The versatility of Profiler has been further demonstrated through its successful application to the analysis of transcriptomic and EEG datasets.

### 3.1 Data conversion and importation

To accommodate vendor heterogeneity, Profiler integrates a vendor-agnostic data conversion module using msconvert from proteowizard (Kessner *et al.* 2008). It supports the conversion of raw files from Bruker, Thermo Fisher, and Waters instruments into open formats such as. mzML, mzXML, .mz5, and .mzDB via pyOpenMS. During conversion, users can: define mass range boundaries, enable peak picking, apply lock mass corrections, downsample spectra for faster processing. This ensures standardization of MS input across platforms and enhances compatibility with downstream tools.

In addition, Profiler accepts and harmonizes a wide variety of omics data types, including MS standard format files, where MS files are structured by biological class or condition using the and parsed using pyOpenMS library13, and tabular omics data in .csv, .tsv, .txt, and .xlsx formats, including exports from MaxQuant (Tyanova *et al.* 2016a), DIA-NN (Demichev *et al.* 2020), and Perseus (Tyanova *et al.* 2016b). The expected format for tabular data requires a column named “Class” for target labels (e.g. control, condition 1, etc.) and the remaining columns as features (ions, gene names, protein names, metabolites, etc.). Additionally, Profiler supports survival and clinical data, requiring “Overall Survival” and “State” columns to facilitate survival modeling and stratification using the lifelines library. Uploaded datasets are automatically cataloged, checked for delimiter consistency, and verified for missing or malformed values. Data handling and manipulation are facilitated by the pandas and openpyxl libraries.

### 3.2 Data conversion and importation

An integrated data exploration module enables users to interactively explore and validate their datasets, offering summarization through visualizations of class distributions, missing values information, and sample sizes.

**Figure 2.**
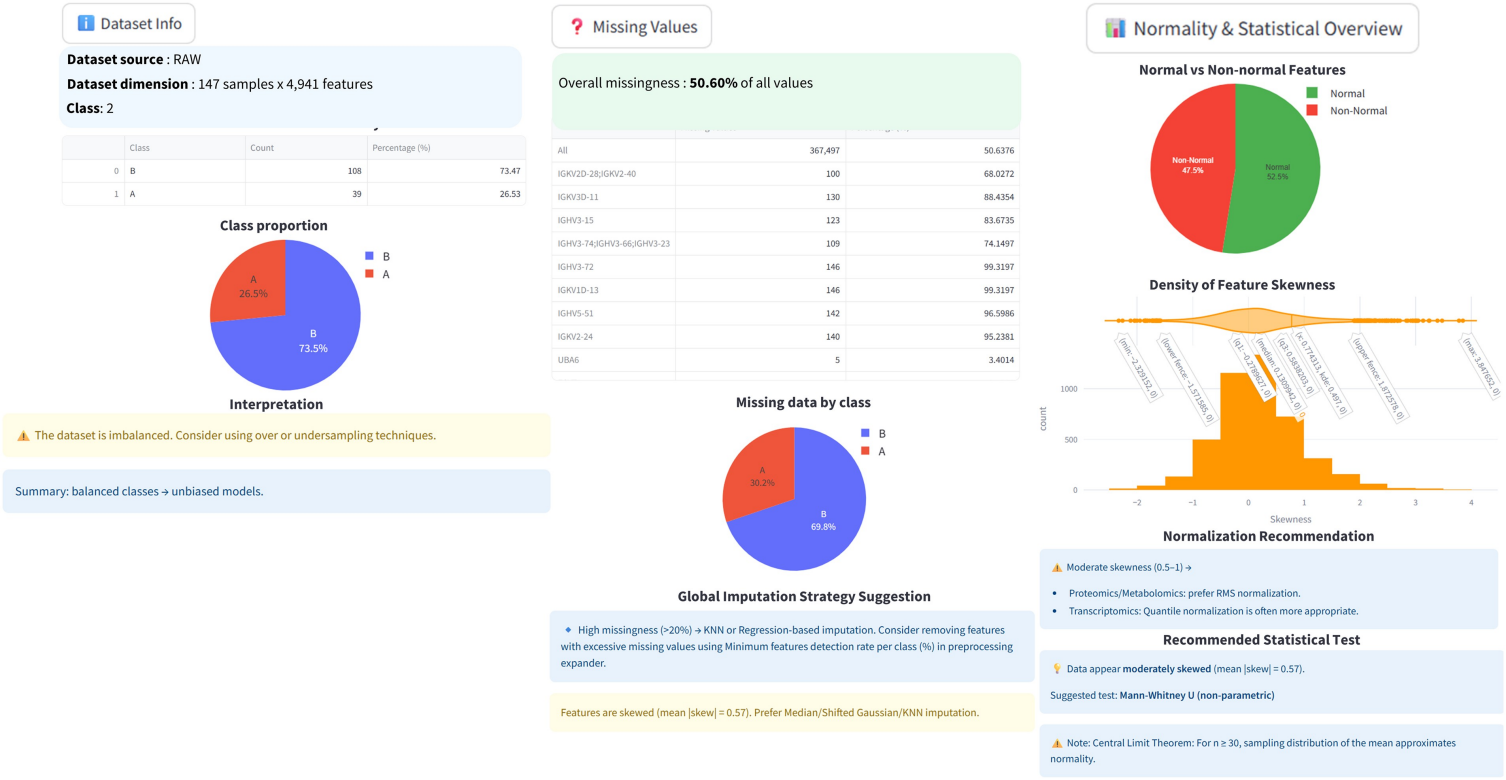
Overview of the insights gained from the data exploration module using Profiler.

As shown in Fig. 2, using the data exploration component of Profiler, the dataset consists of 108 samples in group A (73.5%) and 39 samples in group B (26.5%), indicating a class imbalance that may require over- or under-sampling to address. Furthermore, approximately 50% of the data contains missing values, with a higher proportion in group B. Only half of the features follow a normal distribution, suggesting that *K*-nearest neighbors (KNN) imputation is suitable for handling missing data, and that either the *t*-test or the Mann–Whitney *U* test should be used to assess the statistical significativity depending on the distribution of each variable (feature).

Users can also manage labels by editing class names in-session for clarity and consistency. One module provides various preprocessing options, including normalization techniques such as TIC, RMS, BasePeak, QNorm, and log transformations, as well as batch effect correction using NeuroCombat from the pycombat package (Behdenna *et al.* 2023). Dynamic binning can be applied to selected mass ranges, and missing value imputation is supported through mean, median, mode, and KNN-based imputation using scikit-learn’s KNNImputer libraries (Aljrees 2024).

For our dataset, KNN imputation with missing value removal was used to optimize the dataset and the rest of the data analysis, as it is recommended in Fig. 2. Indeed, given that the dataset contains a balanced mix of values with uncertain distribution characteristics, it is unclear whether mean or median imputation would be optimal. As a result, KNN imputation emerges as the most robust and adaptive solution. Thanks to KNN imputation and the removal of missing values (exclusive features), the total number of proteins falls from 4936 to 4251.

### 3.3 Class balancing and sampling

Profiler includes advanced resampling strategies to correct class imbalance, either by data augmentation or data decrease, which is crucial for training classification models. These strategies include over-sampling techniques such as SMOTE (Synthetic Minority Over-sampling Technique) and ADASYN (Adaptive Synthetic Sampling), which generate synthetic samples to balance the classes and under-sampling techniques like RandomUnderSampler and NearMiss, which reduce the number of samples in the majority class. These resampling methods are applied through the imbalanced-learn library (Lemaıtre and Nogueira xxxx), ensuring full compatibility with structured data and MS intensities, thereby enhancing the performance and reliability of classification models.

In our dataset, applying over-sampling, as recommended in Fig. 2, to address class imbalance would result in 108 samples per group. All subsequent analyses could then rely on this balanced dataset, if wanted.

### 3.4 Data visualization

The visualization engine relies on Plotly, Matplotlib, and Seaborn to generate interactive plots, offering a variety of visualization options and providing a comprehensive and interactive way to explore and understand the data. These include feature distributions displayed through line, bar, histogram, and radar charts, as well as spectra visualization with mean signal/features and individual sample. UpSet and Venn diagrams are used to show the overlap of features across classes, using the upsetplot library and custom logic (Lavanya *et al.* 2023).

**Figure 3.**
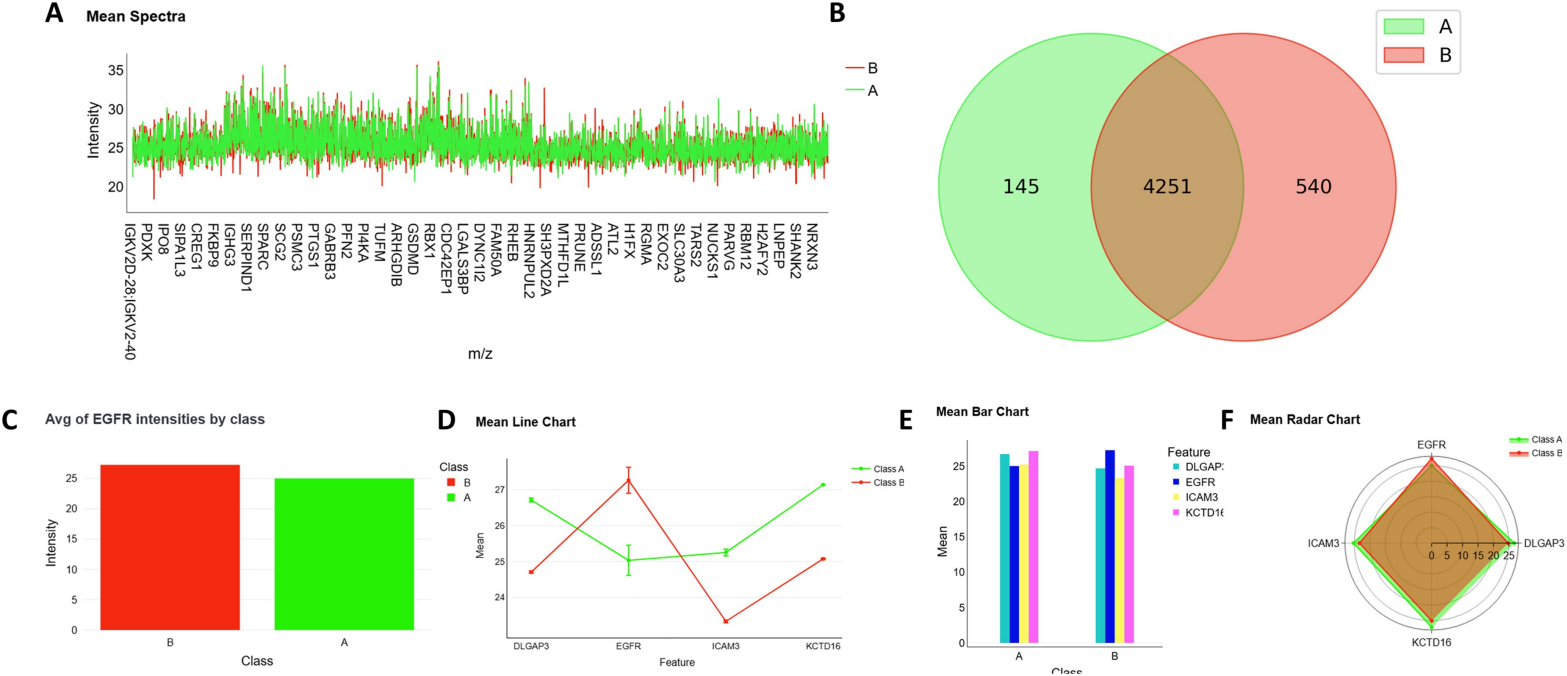
Key findings from the data visualization module using Profiler. (A) Pseudo-spectra displaying the LFQ intensities of all detected proteins across groups. (B) Venn diagram allowing to discover exclusive proteins of each group. (C) Bar chart showing the distribution of a specific protein (EGFR) across different groups. (D–F) Comparison of multiple proteins (EGFR, KCTD16, ICAM3, and DLGAP3) across groups using line plots, bar charts, and radar charts.

Spectra from classical MS datasets can be displayed and interactively explored (Fig. 2, available as supplementary data at *Bioinformatics* online), allowing zooming and other manipulations. In addition, pseudo-spectra, such as the one shown in Fig. 3A, can be visualized to display the LFQ intensities of all detected proteins across groups.

Using the raw data, before applying KNN imputation by Class and removing class-exclusive features (which cannot be imputed as they are not detected in the other class), a Venn diagram can be generated to identify group-exclusive proteins (Fig. 3B). In our case, 145 proteins were found to be exclusive to group A, and 540 to group B, with 4251 proteins in common. However, the exclusive proteins can only be used for pathway enrichment analysis (as presented in the following sections of the article), but not for statistical testing or ML/DL model training. Therefore, for all subsequent analyses, except pathway enrichment, the results rely exclusively on the dataset with no exclusive features and no missing values as they are imputed.

Before performing statistical tests, it is important to explore and better understand the data. Several types of visualizations are available for this purpose. For example, a bar chart can be used to show the distribution of a specific protein across different groups by displaying its presence or absence (Fig. 3C). The protein EGFR, for instance, appears to be more expressed in group B. It is also possible to compare multiple proteins simultaneously using radar charts, line plots, or bar charts (Fig. 3D–F). These visualizations reveal, for example, that EGFR is more abundant in group B, whereas DLGAP3, ICAM3, and KCTD16 are more highly expressed in group A.

### 3.5 Correlation and similarity analysis

To explore inter-feature or inter-class relationships, Profiler offers advanced modules that support exploratory biological hypotheses and quality control. Users can assess intra-feature relationships through correlation methods, including Pearson and Spearman, which are computed between the average feature vectors of each class. Pearson correlation is ideal for normally distributed data, measuring linear relationships, while Spearman correlation is suitable for non-parametric data, assessing monotonic relationships using rank values.

Additionally, inter-class resemblance is evaluated using cosine similarity and Cohen’s Kappa score. Cosine similarity measures the angle between feature vectors of each class, indicating the directional alignment of the data (with 1 signifying identical direction and 0 orthogonal). Cohen’s Kappa, on the other hand, evaluates the agreement in categorized feature profiles after discretizing continuous data into ranked categories (e.g. low, medium, high expression). This discretization allows Kappa to measure agreement on patterns rather than exact numerical values, providing insights into the consistency of feature profiles across classes. These techniques are crucial for understanding the underlying data structure and ensuring the reliability of biological interpretations and the novel application of Cohen’s Kappa within Profiler is particularly valuable for omics analysis, as suitable to reveal consistent expression trends that may be masked by variability at the continuous level.

Figure 3, available as supplementary data at *Bioinformatics* online, shows that while group A and group B are highly correlated (*r* = 0.93), indicating strong similarity in continuous variables, their moderate agreement on Cohen’s Kappa (*κ* = 0.57) suggests notable differences when categorical aspects are considered.

### 3.6 Machine learning and Deep learning

Profiler supports comprehensive ML and DL workflows through scikit-learn, TensorFlow, and custom wrappers, offering a wide range of techniques for both unsupervised and supervised learning.

**Figure 4.**
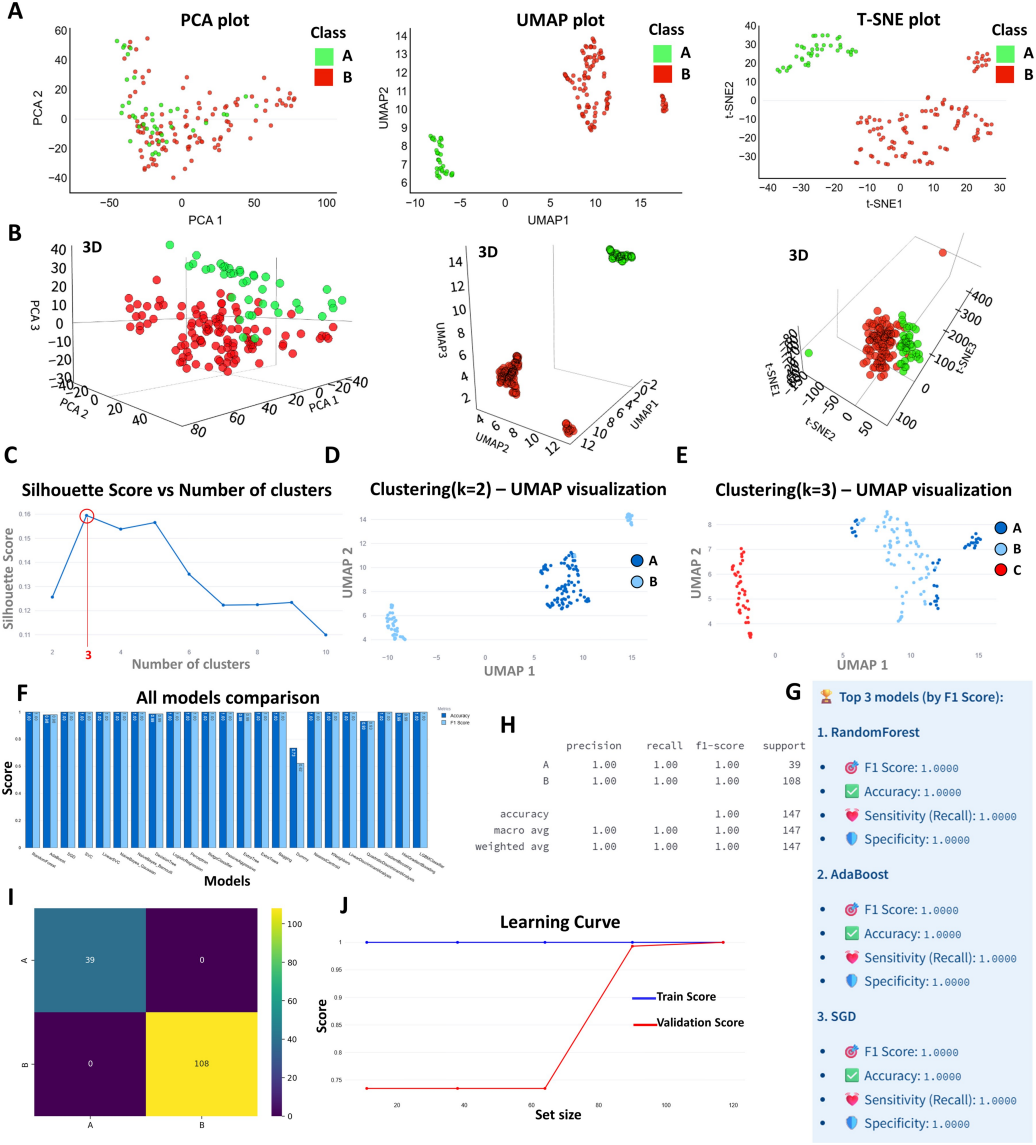
Overview of outcomes generated by the AI modeling module within Profiler. (A, B) 2D and 3D plots of PCA, UMAP, and t-SNE visualizations separating classes A and B. (C) Silhouette scores for different cluster numbers, with the optimal number being 3. (D, E) UMAP clustering results for *k* = 2 and *k* = 3. (F–J) Model accuracies are compared, top three models, classification metrics, confusion matrix, and learning curve for the best-performing model is detailed.

For unsupervised learning, users can employ dimensionality reduction methods such as PCA (principal component analysis), UMAP (Uniform Manifold Approximation and Projection), and t-SNE (t-Distributed Stochastic Neighbor Embedding) to visualize data clusters. The plots can be generated in both 2D and 3D, depending on the dimensionality reduction method used. In complex omics datasets such as proteomics, relationships between variables are typically non-linear. Consequently, PCA, which captures only global linear variance, fails to clearly separate two groups A and B (Fig. 4A), as it does not adequately model the underlying non-linear structure of the data. In contrast, non-linear techniques such as UMAP and t-SNE better preserve local neighborhood relationships and non-linear patterns, resulting in well-separated clusters that distinctly differentiate the two groups (Fig. 4B and C).

For UMAP, the n_neighbors parameter is crucial as it defines the size of the local neighborhood used for manifold approximation. Choosing this parameter can be challenging for scientists, as it is not well-documented and can lead to misleading biological conclusions if not set correctly. To address this, Profiler uses a heuristic approach to calculate n_neighbors based on the number of data points. This heuristic ensures that the neighborhood size adapts to the dataset size, balancing between capturing local structure and computational efficiency. This approach is based on recommendations from the original UMAP paper (McInnes *et al.* 2020) and practical guidelines from the ML community. For t-SNE, the perplexity parameter influences the number of nearest neighbors that are used in other data points. Similar to UMAP, selecting an appropriate perplexity value can be non-trivial and may result in incorrect interpretations if done manually. Profiler calculates perplexity using a heuristic approach based on the number of data points. This heuristic aims to find a balance between preserving local and global data structures while avoiding overfitting. This method is inspired by the original t-SNE paper and best practices in the field. By automating the selection of these parameters, Profiler helps users avoid potential pitfalls and ensures more reliable and reproducible results.

In addition, *k*-means clustering and silhouette analysis (Rousseeuw 1987) can be used to assess group formation and heterogeneity. Indeed, for *k*-means clustering, determining the optimal number of clusters is a critical step. Profiler uses silhouette analysis to evaluate the quality of the clustering. The silhouette score measures how similar an object is to its own cluster compared to other clusters. A higher silhouette score indicates better-defined clusters. By analysing the silhouette scores for different numbers of clusters, Profiler helps users identify the optimal number of clusters without overclustering or underclustering. This ensures that the clustering results are meaningful and biologically relevant.

Looking at our dataset, Silhouette analysis indicates that an optimal clustering would involve three groups, rather than the current two-group classification (A and B) (Fig. 4C). This is consistent with the previous t-SNE and UMAP plots, where two distinct subgroups can be observed within group B, suggesting underlying heterogeneity. This observation is further supported by the UMAP projection with three clusters, where group B clearly subdivides into two separate clusters, referred to as groups B and A, whereas previous group A referred to group C in red (Fig. 4D and E). This suggests that group B may contain multiple tumor clones or distinct subtypes. In previous lipid-MSI studies, patients from group B often showed high levels of necrosis, which could also explain the observed heterogeneity. To explore this further, integrating additional clinical metadata, such as age, sex, treatment history, or comorbidities, could help identify meaningful biological or clinical differences and improve patient stratification.

In supervised learning, Profiler provides access to over 23 models, including random forest, logistic regression, SVM, naïve Bayes, gradient boosting, and LDA/QDA, along with ensemble methods like bagging classifiers. Users can compare model performance using learning curves, confusion matrices, and classification reports with metrics such as *F*1 scores, accuracy, recall, precision, sensitivity, and specificity.

When attempting to build the optimal classification model using our dataset, 20 out of the 23 tested algorithms reached perfect accuracy (100%) after 20-fold cross-validation (Fig. 4F and G). This performance underscores a clear separation between the groups and indicates that the models successfully captured distinct protein profiles characteristic of each group. Notably, both the confusion matrix and the classification report demonstrate that the optimal model, built using the RidgeClassifier algorithm, achieved perfect performance with no misclassifications (Fig. 4H and I). The learning curve shows that the model begins to learn effectively after 70 samples and reaches optimal performance by 90 samples. Furthermore, the close alignment of the training and validation curves toward the end indicates good generalization, with no apparent underfitting or overfitting (Fig. 4J).

For DL, Profiler supports architectures like MLP (multilayer perceptron), CNN (convolutional neural network), and RNN (recurrent neural network), with accelerated training and real-time metric tracking. DL typically requires large amounts of data to be truly effective. In our case, the dataset is not extensive enough to provide a clear advantage over traditional ML approaches. Nevertheless, as shown in Fig. 4, available as supplementary data at *Bioinformatics* online, the DL algorithms (MLP and CNN) still managed to achieve 100% accuracy in classifying the two groups.

**Figure 5.**
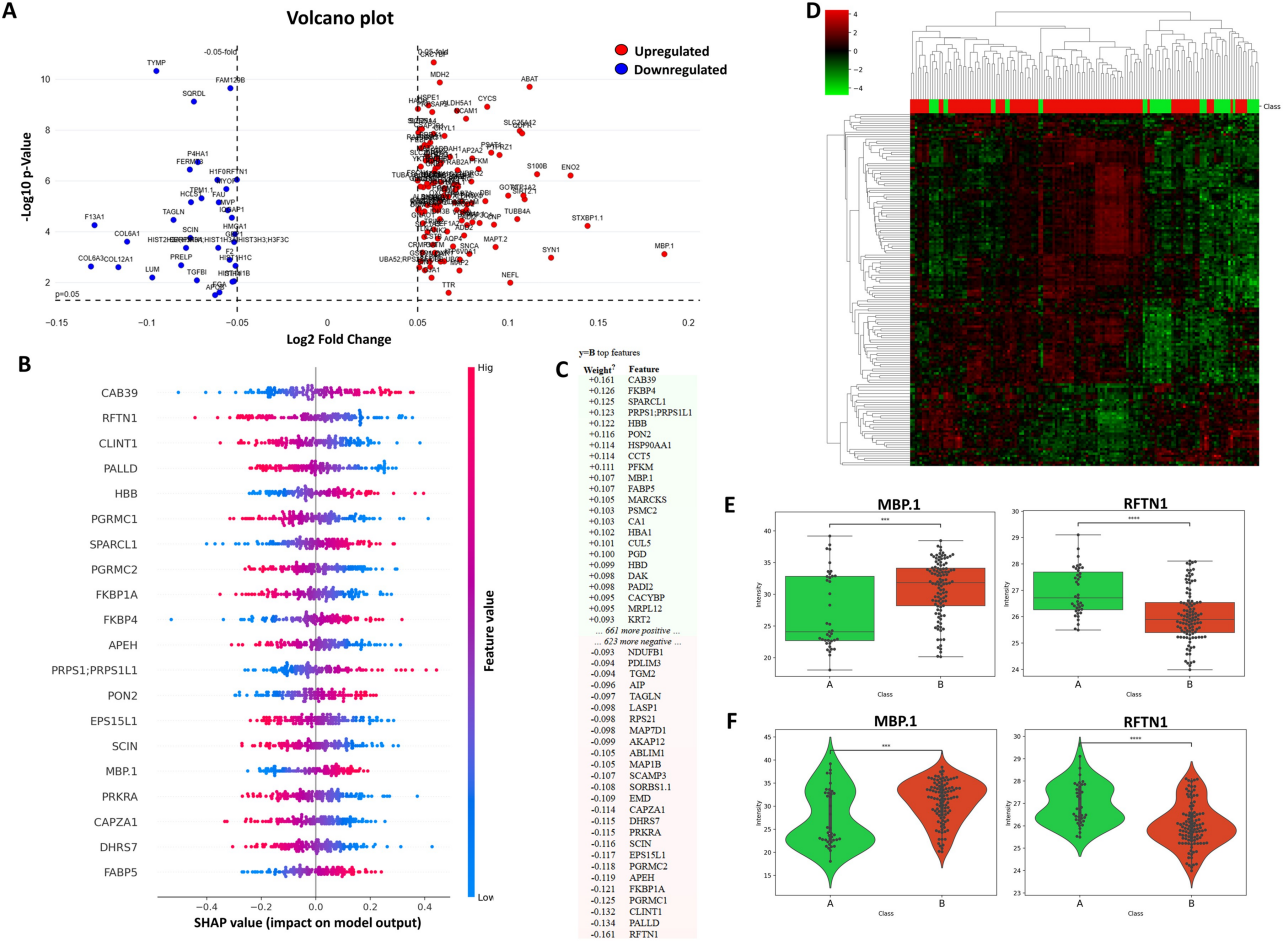
Overview of the outcomes generated by the Profiler-based biomarker discovery module. (A) Volcano plot with a 0.05-fold change threshold and *P*-value of .05, showing up- and down-regulated proteins depending on the groups. (B) SHAP beeswarm plot of the top 20 proteins contributing to the ML model. (C) LIME analysis showing the top 46 proteins contributing to the ML model. (D) Heatmap clustering the two groups based on deregulated proteins identified by both the volcano plot and AI explainability methods. (E, F) Boxplots and violin plots displaying the expression levels of two deregulated proteins (MBP.1 and RFTN1).

**Figure 6.**
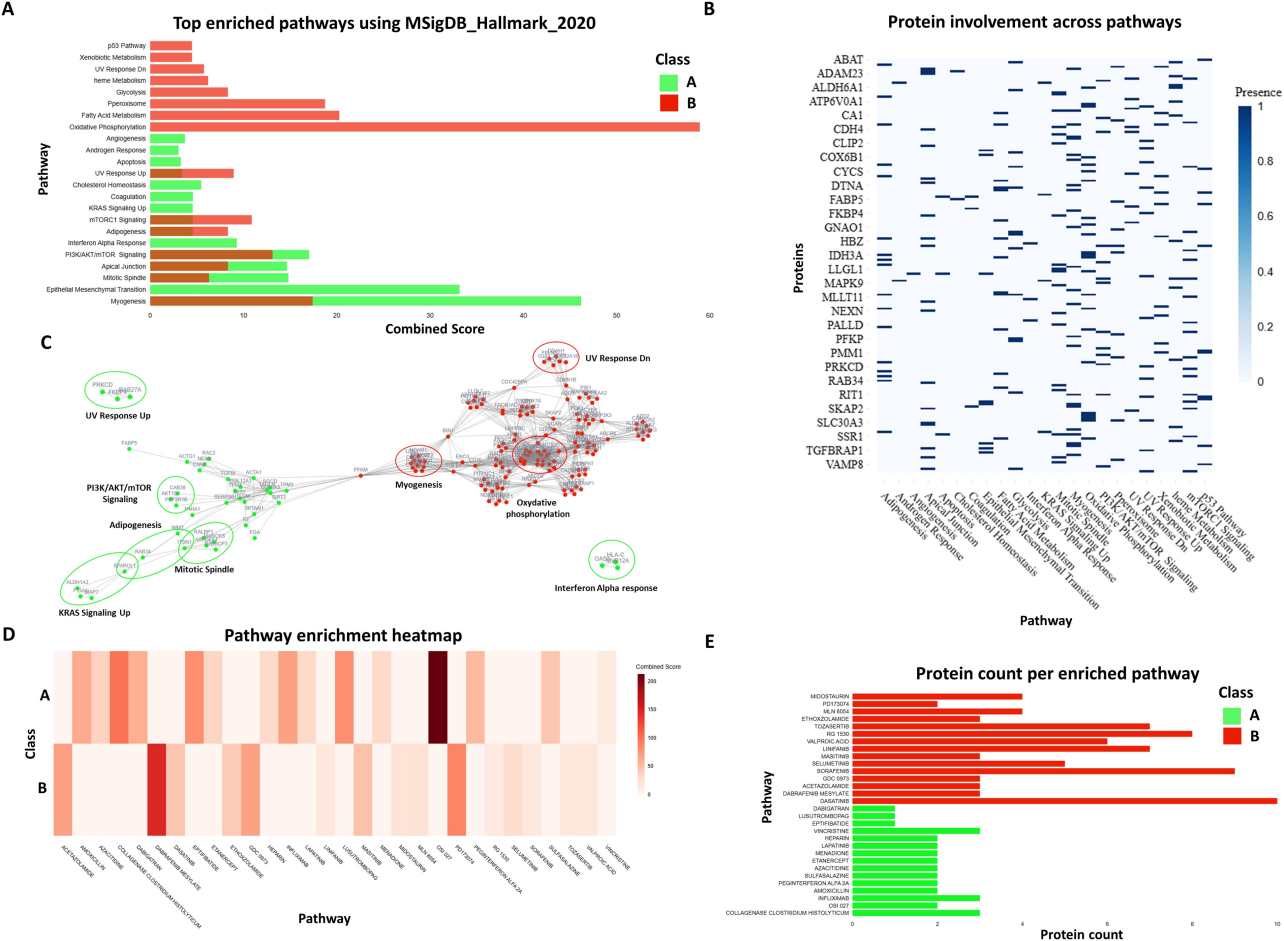
Pathways and drugs enrichment using enrichment module in Profiler. Panels with (A) enriched pathways bar plot according to combined score, (B) gene involvement across pathways depicted as an heatmap, (C) interactive gene interaction network, (D) enriched drugs heatmap according to combined score, and (E) enriched drugs according to gene count.

To provide a more quantitative comparison between approaches, we included a benchmarking summary (Table 2 and Figs 5 and 6, available as supplementary data at *Bioinformatics* online) comparing representative ML and DL models in terms of accuracy, *F*1-score, and training time, using identical cross-validation settings. The results confirm that classical ML algorithms achieved comparable or slightly higher performance (max accuracy of 0.95) with substantially shorter training times, whereas DL models such as MLP and CNN required longer optimization but reached similar accuracies (max accuracy of 0.91). This comparison highlights that ML approaches remain highly efficient and competitive for moderate-sized omics datasets, while DL methods are expected to show greater advantages as dataset size and heterogeneity increase.

Users can save and reload trained models along with the selected features, the fitted label encoder and the full preprocessing pipeline, including scaling and transformations. This ensures that any new data used for prediction will undergo the exact same preprocessing steps as the training data, maintaining consistency and avoiding data leakage. Importantly, saving the specific trained features (not just the input dimension) guarantees that the model only processes the variables it was originally trained on, preserving both model integrity and performance. This is particularly crucial when applying the model to new omics data, such as metabolomic spectra, proteomic LFQ, or gene/RNA expression, where some features used during training may not be detected in a given sample. In traditional workflows, this mismatch would prevent prediction altogether. However, Profiler handles this seamlessly by assigning a default value (e.g. zero) to any missing feature, treating it as not detected. This allows predictions to proceed using the available features without compromising model compatibility or requiring retraining.

This approach enhances reproducibility, ensures robust and interpretable predictions, and supports scalable deployment in real-world scenarios. All models can be exported for external use, making Profiler a powerful and flexible tool for both exploratory analysis and predictive modeling across diverse omics applications.

### 3.7 Biomarker discovery

Next, Profiler offers a comprehensive pipeline for biomarker discovery and feature interpretation, which also serves as a robust feature selection process. This pipeline includes a variety of statistical analysis tools and explainability modules designed to identify, rank, and visualize significant biomarkers. These insights can then be saved as structured dataframes for further analysis or model retraining, enhancing overall performance and interpretability.

One of the standout features is the volcano plot, conventionally used to compare binary classes. However, Profiler has expanded this functionality to support multi-class comparisons, providing a more versatile tool for biomarker discovery. Volcano plots visualize the statistical significance (*P*-value) and magnitude of change (fold change) for each feature, allowing users to quickly identify the most relevant biomarkers. Provides also option to highlight feature names for better clarity and offers a features detection based on intensity thresholds, which can automatically identify and include significant features in the analysis. This multi-class capability broadens the applicability of volcano plots, making them a powerful tool for complex datasets.

For the biomarker discovery analysis, the dataset used was processed using KNN imputation with a 70% presence threshold, ensuring that only proteins detected in at least 70% of samples per class were retained. This filtered dataset was used for the generation of the volcano plot and heatmap, as well as employed for model training (Fig. 6A, available as supplementary data at *Bioinformatics* online), used for the computation of SHAP and LIME values.

Using a volcano plot with a 0.05-fold change, a .05 *P*-value and FDR Benjamini–Hochberg as multiple testing correction method, 145 proteins were found significantly deregulated in group A or B (Fig. 5A).

Profiler also integrates explainability tools to enhance the interpretability of ML results. It supports SHAP (SHapley Additive exPlanations) (https://shap.readthedocs.io) for both local and global attribution (Lundberg and Lee 2017), and LIME (https://eli5.readthedocs.io) for introspection of models. SHAP values provide detailed explanations of model outputs by quantifying the contribution of each feature to individual predictions, offering both per-sample and overall insights. LIME, on the other hand, offers transparency in models by highlighting feature weights/contributions and their effects (positive or negative). Profiler includes custom modules that convert SHAP and LIME outputs into structured DataFrames, facilitating easier downstream analysis and integration. In addition, various visualization techniques such as beeswarm plots and positive/negative contribution plots are generated to visually feature impacts and enhance understanding of model behavior. Together, these tools ensure that predictive models are not only accurate but also trustworthy and explainable.

Using AI explainability tools, 24 proteins that contributed most to the model’s predictions were identified (Fig. 5B and C) (Fig. 6B, available as supplementary data at *Bioinformatics* online). These proteins were added to those found deregulated in the volcano plot, except when already recurrent such as MBP.1 for group B and RFTN1 for group A, for further analysis.

Additionally, Profiler offers heatmap clustering for both features and samples, enabling users to visualize patterns and relationships within the data. Users can perform heatmap clustering on all or selected features, with options to average feature values by class and apply statistical tests to filter significant features. Customizable parameters include the choice of data type (original intensity or log2 transformed) and *P*-value thresholds, allowing for tailored analysis. The heatmaps are enhanced with custom color schemes to highlight under-expression, neutral expression, and over-expression, providing a clear and intuitive visualization.

A heatmap generated using all 169 discovered biomarkers (125 upregulated in group B and 44 in group A), from both volcano plots and AI explainability methods, clearly demonstrated a strong clustering of the two groups, with distinct patterns of under- and overexpressed proteins (Fig. 5D). Moreover, when comparing the heatmaps generated using the biomarkers from the volcano plot and those identified through AI, we observe that the one derived from volcano plot appears to be clustered in a much more homogeneous manner (Fig. 7B, available as supplementary data at *Bioinformatics* online). In contrast, the heatmap based on AI biomarkers shows a noticeable heterogeneity, particularly within group B (Fig. 7A, available as supplementary data at *Bioinformatics* online).

For statistical analysis, Profiler supports a range of tests tailored to both binary and multi-class scenarios, including parametric and non-parametric methods. Users can perform *t*-tests and ANOVA for parametric data, as well as Kruskal–Wallis and Mann–Whitney tests for non-parametric data. These tests help assess the significance of features and their correlation with biological conditions by facilitating the visualization using boxplots, violin plots, or bar plots.

Here, two examples of deregulated proteins were displayed using boxplots and violinplots (Fig. 5E and F) using Kruskal–Wallis test. Indeed, it showed that in a significantly manner, MBP.1 is overexpressed in group B, in contrary to RFTN1 who is more expressed in group A.

Overall, Profiler’s biomarker discovery and feature interpretation pipeline is designed to streamline the process of identifying significant features, enhancing model performance, and providing clear, interpretable results. The ability to save these insights as structured dataframes further facilitates downstream analysis and model retraining, ensuring that users can leverage the most relevant features for their research.

### 3.8 Pathway enrichment and functional annotation

Biological pathway analysis in Profiler is performed using GSEApy (Fang *et al.* 2023), interfaced via custom algorithm. This feature allows users to select from multiple comprehensive databases, more than 100 databases, including KEGG (Kanehisa and Goto xxxx), GO (The Gene Ontology Consortium 2015), Reactome (Fabregat *et al.* 2018), MSigDB (Liberzon *et al.* 2011), and Drug Signatures (Barrett *et al.* 2013, Svoboda *et al.* 2019), providing a wide range of biological contexts for analysis. One of the key advantages of Profiler is its support for multi-class enrichment, which facilitates comparative insights across different phenotypes. This is particularly useful for studies involving multiple conditions or treatments, as it allows for a more nuanced understanding of biological pathways. For each pathway, Profiler provides detailed information including the number of associated proteins or genes, as well as the list of implicated features within that pathway. Importantly, Profiler also highlights genes or proteins that are not associated with any enriched pathways, allowing users to capture the full scope of molecular involvement, including potentially novel or understudied factors. The results of the enrichment analysis are visualized in enriched term graphs, heatmaps, and interactive plots, which provide an intuitive way to explore the significance of various pathways. Additionally, the results can be exported as structured tables, making it easy to integrate the findings into further analyses or reports.

By using all identified biomarkers (exclusive features, volcano plots feature selection, and markers highlighted via AI explainability) and applying the enrichment module, we identified the top 15 enriched pathways for each group using the MSigDB_Hallmark_2020 database. These pathways were ranked based on their combined score and visualized as either bar plots (Fig. 6A) or heatmaps (Fig. 8A, available as supplementary data at *Bioinformatics* online), or based to features counts (Fig. 8B, available as supplementary data at *Bioinformatics* online). In addition, the specific proteins involved in each enriched pathway can be retrieved (Fig. 6B) (Data 1, available as supplementary data at *Bioinformatics* online). Even more interestingly, their interaction network is visualized in Fig. 6C, revealing complex interactions within and between certain pathways. This analysis revealed, e.g. that group A tumors are enriched in pathways such as myogenesis (cell differentiation) and interferon alpha response (antiviral immune response). Overall, group A appears to activate differentiation, immune response, tissue integrity, and signaling, suggesting a more stable, less invasive, and potentially less aggressive tumor phenotype. In contrast, proteins in group B are involved in intense metabolic activity, oxidative stress, and p53 response pathways, which are characteristic of aggressive and proliferative tumors. This suggest that these tumors are rapidly growing and potentially more resistant to treatment, leading to shorter survival.

Going further, we explored potential drug targets using the DGIdb_Drug_Targets_2024 database to identify compounds that could specifically target the previously enriched pathways in each group. As with the pathway analysis, the results were visualized using multiple plot types (Fig. 6D and E) (Fig. 8C and D, available as supplementary data at *Bioinformatics* online). Group B shows strong enrichment in oncogenic kinase inhibitors such as Dabrafenib, Dasatinib, and Masitinib, targeting pathways like MAPK and BRAF that drive cell proliferation and therapeutic resistance. This pattern indicates hyperactivation of proliferative signaling, consistent with a more aggressive and resistant tumor phenotype. In contrast, group A is enriched in immunomodulatory and anti-inflammatory drugs (e.g. Infliximab, Etanercept, Sulfasalazine) along with anticancer agents such as Lapatinib and OSI-027. This suggests a distinct, less aggressive profile, potentially more responsive to immune modulation and conventional therapies (Data 2, available as supplementary data at *Bioinformatics* online). Indeed, the enrichment analyses are exploratory and aim to highlight potential biological relationships between drug-associated targets and the studied gene/protein classes.

The enrichment-based identification of drug–target associations should be regarded as hypothesis-generating. Computational drug repurposing approaches, while powerful, are limited by target promiscuity, context-dependent activity and incomplete pathway annotation (Pushpakom 2019). Future validation will require experimental confirmation, for instance, through *in vitro* binding or cell-based functional assays, as emphasized in recent repurposing frameworks (Corsello *et al.* 2017, Pushpakom *et al.* 2019).

Profiler also offers an interactive gene interaction network using NetworkX, a powerful Python library for the creation, manipulation, and study of complex networks. This network visualization allows users to explore the relationships between genes involved in enriched pathways, providing deeper insights into the biological mechanisms at play. Users can dynamically interact with the network, zooming in on specific genes or pathways to understand their connectivity and importance. The network is color-coded based on the protein type or class, making it easy to distinguish between different groups of genes. This interactive feature enhances the interpretability of the enrichment results and helps researchers identify key genes and pathways that may be crucial for further investigation.

### 3.9 Survival and prognostic modeling

Using the lifelines library (Davidson-Pilon 2019), Profiler supports advanced survival and prognostic modeling techniques. Key features include Kaplan–Meier estimation, which provides a non-parametric way to estimate the survival function from lifetime data, and Cox proportional hazards modeling, which assesses the effect of several risk factors on survival time. Additionally, Profiler supports log-rank tests to compare the survival distributions of two or more groups. These tools are essential for translational biomarker studies, where understanding the prognostic value of various covariates is crucial. By integrating these survival analysis techniques, Profiler enables researchers to identify factors that significantly impact survival outcomes, aiding in the development of more effective treatment strategies and personalized medicine approaches. Furthermore, the Cox proportional hazards model can be saved and deployed directly within Profiler to make predictions on new data or patients, facilitating real-time prognostic assessments and enhancing clinical decision-making.

Our analysis used Kaplan–Meier survival curves and a Cox proportional hazards model to assess survival outcomes and influencing factors for the two distinct groups, A and B. Figure 9A, available as supplementary data at *Bioinformatics* online, with Kaplan–Meier curves, reveal a significant survival advantage for group A over group B, with a *P*-value of .00001, indicating this difference is statistically significant. Figure 9B, available as supplementary data at *Bioinformatics* online, with a forest plot from the Cox model, identifies key proteins impacting survival, with log (hazard ratios) and 95% confidence intervals illustrating their effects (Data 3, available as supplementary data at *Bioinformatics* online). Indeed, variables to the right of zero indicate increased hazard and worse survival, while those to the left suggest better survival prospects. We can observe, for instance, that the protein HBB (hemoglobin subunit beta) is associated with shorter survival, and act as a negative prognostic factor (Gautam *et al.* 2012, Ponzetti *et al.* 2017). In contrast, CAPZA1 (capping actin protein subunit alpha 1) is associated with longer survival, suggesting a protective role (Li *et al.* 2021). This could be explained by a role in limiting tumor mobility/migration or in stabilizing cellular architecture, reducing tumor aggressiveness. These observations suggest that proteomic signature captures biologically meaningful differences between the long-survival (Class A) and short-survival (Class B) glioblastoma groups. However, as with any observational model, validation in independent cohorts and experimental follow-up are needed before clinical translation.

### 3.10 Wizard and deployment tools

Wizard module designed to guide users through real-time and *post hoc* prediction workflows, enhancing the accessibility and utility of predictive modeling. This module supports real-time predictions on new samples directly from raw files, a feature initially designed for real-time prediction connected to mass spectrometer instruments. While real-time prediction directly from the instrument is not feasible when using Profiler from the web, users can still achieve real-time predictions by dragging and dropping a zipped raw file from instruments such as Waters, Bruker, or Thermo. This capability ensures that users can leverage Profiler’s predictive power even in environments where direct instrument integration is not possible. Additionally, the wizard module facilitates post-hoc predictions using tabular datasets and saved models. Users can upload tabular data and apply saved models to make predictions, with (“Class” column) or without ground truth data. This flexibility allows for the comparison and assessment of test datasets against known outcomes, providing valuable insights into model performance. The results of these predictions can be visualized, interpreted, and exported in publication-ready formats, making it easy to share findings with colleagues or include them in research publications.

Using the same dataset employed for spectral visualization in a previous module, originating from the study by Zirem *et al.* (2024a,b), a classification model was built, achieving 92% accuracy through five-fold cross-validation. This model was then tested blindly on an unseen dataset using the wizard module of Profiler. Two ways of predictions are available, either using a raw data (real-time or post-acquisition way) or using an already processed csv file (*post hoc* way). As shown in Figs 10 and 11, available as supplementary data at *Bioinformatics* online, the real-time predictions were highly satisfactory, with no misclassifications.

A novel and powerful feature introduced in Profiler is the ability to simultaneously interrogate multiple trained models. Users can upload several models, with the same trained features and label encoders, and Profiler will perform predictions using all models in parallel. The final class is then determined by a majority voting strategy and a confidence score is provided to reflect the consensus across models. This ensemble-like approach improves prediction robustness, compensates for model-specific biases and ensures more reliable decision-making in practical applications.

### 3.11 Multi-omics capabilities of profiler

In order to demonstrate Profiler’s multi-omics capabilities, the various modules presented above, demonstrated on proteomic or lipidomic data, were also used on transcriptomic and electroencephalogram (EEG) data.

Profiler was first tested on a transcriptomic dataset to assess its ability to distinguish between five cancer types: breast invasive carcinoma (BRCA), kidney renal clear cell carcinoma (KIRC), colon adenocarcinoma (COAD), lung adenocarcinoma (LUAD), and prostate adenocarcinoma (PRAD). This dataset originates from the UCI Machine Learning Repository (“Gene Expression Cancer RNA-Seq” dataset), which is derived from the RNA-Seq (HiSeq) PANCAN collection (Fiorini 2016). It consists of randomly selected gene expression profiles from patients diagnosed with these five tumor types. In total, the dataset comprises 801 samples and 20 531 genes.

As shown in Fig. 12, available as supplementary data at *Bioinformatics* online, the three dimensionality reduction algorithms, UMAP, PCA, and t-SNE, successfully distinguished the five cancer types, with UMAP and t-SNE providing better separation. The training and cross-validation of multiple ML models further demonstrated Profiler’s ability to handle large-scale transcriptomic datasets, with more than 13 algorithms achieving 100% classification accuracy after five-fold cross-validation. Additionally, the volcano plot highlights Profiler’s capability to identify potential genomic markers specific to each cancer type.

Subsequently, Profiler was applied to EEG data from a study designed to diagnose epileptic seizures. For this purpose, the frequency components of the EEG signals were extracted using the discrete wavelet transform and parametric methods based on autoregressive modeling. The dataset comprised 11 500 samples containing 179 s of recorded EEG activity.

As shown in Fig. 13, available as supplementary data at *Bioinformatics* online, both t-SNE and UMAP successfully differentiated patients who experienced epileptic seizures from those who did not. The classification model based on the HistGradientBoosting algorithm achieved 98% accuracy after five-fold cross-validation. Finally, the SHAP explainability analysis identified the specific EEG time segments that contributed most to distinguishing between seizure and non-seizure patients.

### 3.12 Additional tools

To enable seamless integration of MSI data into Profiler, we developed a complementary open-source tool named MSI2Profiler. This utility allows users to preprocess MSI datasets in the imzML format and automatically generate a compatible CSV dataframe ready for import into Profiler. Implemented in Python with a Tkinter-based graphical interface, MSI2Profiler performs extraction, normalization and formatting of MSI pixel data for downstream AI model training and prediction within Profiler. The tool is freely available in the Profiler GitHub repository under the Tools directory, and a screenshot of the interface is provided in Fig. 14, available as supplementary data at *Bioinformatics* online.

## 4 Discussion

The increasing volume and complexity of omics data continue to push the boundaries of computational biology. Tools capable of managing and interpreting such data must not only be powerful and statistically sound but also accessible to the wider research community (Marx 2013). Profiler addresses this need by offering an end-to-end, modular solution that unifies multiple analytical capabilities within a single, web-based application or desktop version for offline use.

Unlike existing platforms such as Galaxy, which require complex installation and server configuration, or Perseus, which is confined to Windows environments, Profiler is platform-independent and lightweight, designed to run efficiently on a wide range of systems. Its web-based architecture ensures broad accessibility, and its scalability is evidenced by its performance on high-capacity. This makes Profiler suitable for both small laboratory experiments and large-scale clinical studies. The results obtained on proteomic, lipidomic, transcriptomic, and EEG datasets illustrate the robustness and versatility of the platform.

A distinguishing feature of Profiler is its seamless integration of ML and DL modules, enabling sophisticated predictive modeling directly from user-uploaded data. By embedding preprocessing, feature selection, model training, and evaluation into an intuitive workflow, Profiler lowers the barrier to entry for advanced data science techniques in biology (Libbrecht and Noble 2015). Furthermore, the inclusion of automated biomarker discovery and survival analysis tools allows for clinically relevant insights to be drawn with minimal overhead.

Another critical advantage lies in the platform’s support for data visualization and interpretability. Profiler offers real-time interactive plots, such as reduction methods (PCA, t-SNE, UMAP), volcano plots, clustering heatmaps, box/violin plots, which are essential for hypothesis generation and exploratory data analysis. Importantly, these visualization and statistical modules natively support both binary and multi-class omics datasets, enabling users to explore complex biological scenarios involving more than two conditions or phenotypes. These features not only improve user engagement but also facilitate deeper understanding of data structure and biological patterns. However, the enrichment analyses and drug–target predictions generated by Profiler should be viewed as hypothesis-generating rather than confirmatory. Computational enrichment cannot substitute for experimental validation, particularly due to target promiscuity and pathway context-dependence. Future work should therefore prioritize experimental validation strategies for the most promising candidates, including *in vitro* target engagement assays to confirm compound–protein binding and organoids-based models. Such experimental validation efforts will be essential to translate computational predictions into biologically and clinically actionable insights.

Future developments will include an explicit multi-omics integration module, enabling joint modeling of complementary data layers (e.g. proteo-lipidomic and transcriptomic fusion). The modular architecture already includes placeholders for such integration, ensuring seamless evolution toward network-based and correlation-based data fusion strategies.

From a software engineering standpoint, Profiler was built with extensibility in mind. Its modular design allows for rapid integration of new analytical methods and data types as the field evolves. Future directions include the incorporation of single-cell omics support, release, and integrates pre-trained models for domain-specific applications such as bacterioscoring, immunoscoring, and dry proteomics. These models, validated in prior peer-reviewed studies (Fabregat *et al.* 2018, Zirem *et al.* 2024, Lagache *et al.* 2025) offer domain-specific scoring pipelines that are seamlessly integrated into the workflow. This fusion of enrichment-driven interpretation with task-specific predictive modeling allows researchers to not only observe differential expression patterns but also contextualize them within a biological or clinical framework, supporting hypothesis generation, validation, and translational impact.

To ensure scalability and maintain user experience, Profiler is currently hosted on the HPC infrastructure of the Mésocentre of Lille, with access to 246 GB RAM, multiple CPUs, and expandable GPU capacity. Should usage statistics indicate high demand, we are prepared to scale up computational resources accordingly by increasing CPU, GPU, and RAM allocations, in collaboration with the Mésocentre’s HPC provisioning team. This commitment ensures that Profiler remains responsive and capable of handling large-scale bioinformatics workflows.

In conclusion, Profiler represents a powerful addition to the bioinformatics toolkit. By combining robust analytics with a user-centered design, it closes a critical gap in omics data analysis. We anticipate that Profiler will serve as a valuable resource for biologists, clinicians, and data scientists alike, accelerating discovery in diverse research areas ranging from cancer genomics to microbial ecology.

## Supplementary Material

btaf644_Supplementary_Data

## Data Availability

Profiler is openly accessible at https://prism-profiler.univ-lille.fr. All datasets used in this study are available in the dedicated GitHub repository at https://github.com/yanisZirem/Profiler_v1_requests_datatests in the data_for_peerReview_paper folder. In addition to the datasets used in the article, the repository includes a wide range of real and simulated datasets designed to showcase Profiler’s capabilities across multiple omics platforms. It contains raw MS data acquired from Bruker and Waters instruments (Bruker_data and Waters_data), as well as processed output files from DIA-NN (DIA-NN_data) and MaxQuant (Maxquant_data). The Tabular_data_multi_omics directory offers structured toy datasets specifically created to help users get started with Profiler, test its different modules, and explore its full potential. These datasets, covering lipidomics, proteomics, transcriptomics, and metabolomics, are tailored for binary classification (e.g. aggressive vs. non-aggressive tumors) and multi-class tasks (e.g. tumor, necrotic, and healthy tissues). Additionally, the Survival_data folder contains clinical variables and lipid markers (clinical_and_LipidsMarkers.csv) for Cox regression modeling, as well as preprocessed patient data (statuts_patients.csv) for Kaplan–Meier survival analysis. All data are shared in accessible formats to encourage transparency, reproducibility, and broader adoption by the scientific and educational communities. Furthermore, the same repository provides a Tools directory that hosts the complementary open-source tool MSI2Profiler. The complete source code of the Profiler platform is available in the GitHub repository (https://github.com/yanisZirem/prism-profiler), which also provides detailed instructions for installing the Desktop version. The code is also archived on Zenodo (https://doi.org/10.5281/zenodo.17478158).
