# Supplementary material for: Profiler: an open web platform for multi-omics analysis
Source: Bioinformatics. 2025 Dec 1;42(1):btaf644. doi: 10.1093/bioinformatics/btaf644 (PMC12784250; doi:10.1093/bioinformatics/btaf644)
Supplement: btaf644_Supplementary_Data [file btaf644_supplementary_data.zip › Supplementary_data_Zirem_2025/Supplementary_figures_ZIREM.pdf]

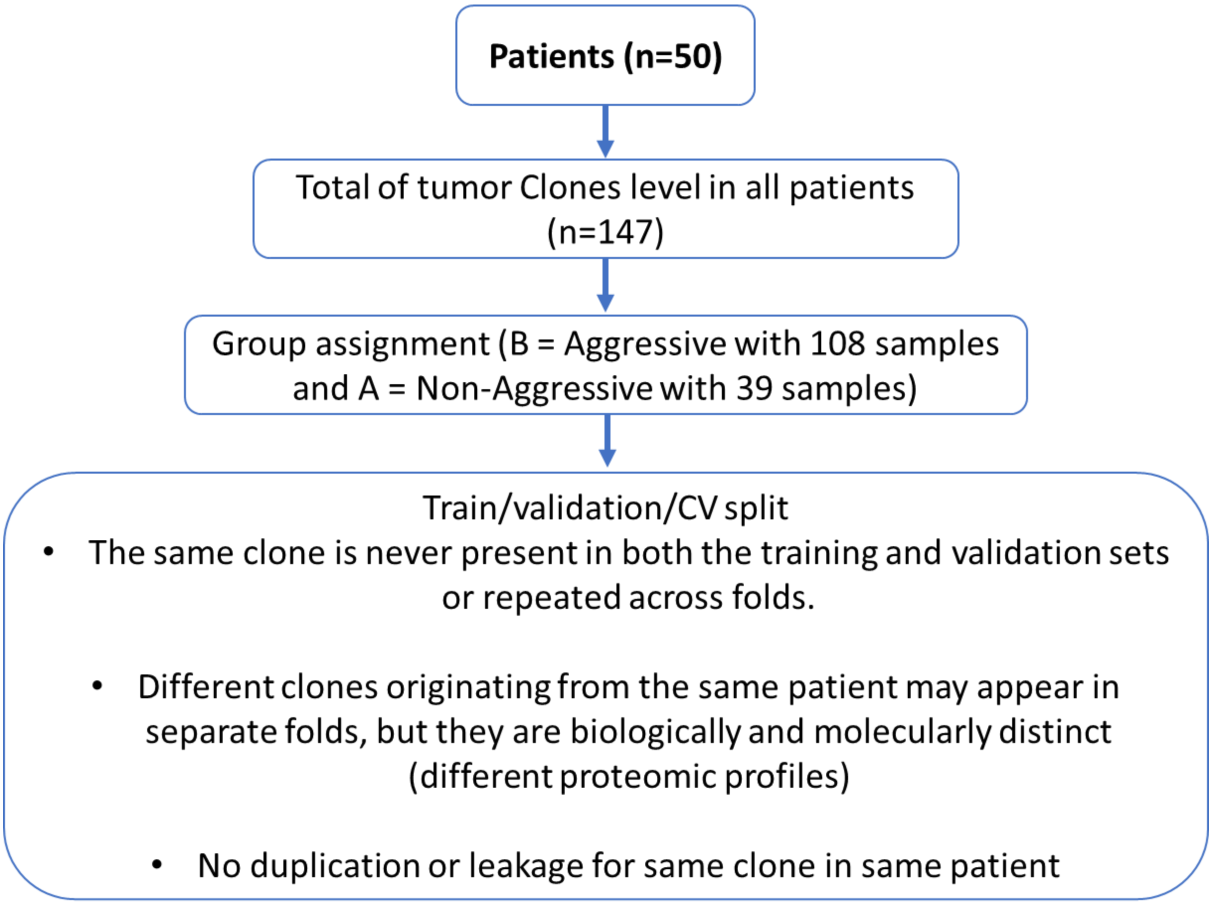

Supplementary Figure 1. Explication of the proteomic dataset used in this article

Supplementary Table 1. Short glossary of key technical terms used in this article.

Term	Definition
Accuracy / F1-score	Common metrics for evaluating classification model performance; Accuracy reflects discrimination power, F1 balances precision and recall
Anonymization	Removal or transformation of identifiers from data to ensure individual privacy and data protection compliance
Batch Effect	Non-biological variation in data due to differences in experimental batches, instrumentation, or processing conditions
Classifier	An algorithm that assigns data samples to predefined categories (e.g., tumor vs. normal)
CNN (Convolutional Neural Network)	Deep learning model that extracts spatial or local patterns, commonly used for image or structured feature data
Cross-Validation	A statistical method (e.g., k-fold) for assessing model performance by repeatedly partitioning data into training and testing subsets
Deep Learning (DL)	A subset of ML using multi-layered neural networks (e.g., CNN, RNN, MLP) capable of learning complex hierarchical features
Dimensionality Reduction	Techniques (e.g., PCA, t-SNE, UMAP) that reduce the number of features while preserving essential data structure and variability
Epoch / Batch Size / Learning rate	Parameters controlling neural network training dynamics
Explainable AI	Methods that make AI and ML model decisions interpretable to humans, using tools like SHAP or LIME
FDR (False Discovery Rate)	Statistical measure indicating the expected proportion of false positives among significant findings
Feature	A measurable variable or attribute used as input for machine learning models (e.g., a protein intensity or gene expression value)
Feature Importance	Quantitative measure of how much each variable contributes to a model's prediction
Feature Selection	The process of selecting the most informative variables from a dataset to improve model performance and reduce noise
imzML	Open file format for storing and sharing MSI data, containing both spectral and spatial information
k-Fold Cross-Validation	Technique where data are divided into *k* subsets (folds) ; each fold is used once for testing and *k–1* times for training
Lipidomics	Study of lipids and their roles in cellular structure, signaling, and metabolism
Machine Learning (ML)	A set of algorithms that learn from data to make predictions or identify patterns without explicit programming
Metabolomics	Analysis of metabolites (small molecules) to characterize metabolic processes and phenotypes
Missing Values	Data points that are absent or undetected in a dataset; they can be handled by imputation, filtering, or statistical modeling
MLP (Multilayer Perceptron)	A fully connected neural network composed of multiple layers of neurons; used for general classification and regression tasks

MSI (Mass Spectrometry Imaging)	Technique that maps the spatial distribution of molecules (e.g., proteins, lipids) directly from tissue sections using mass spectrometry
Multi-omics	Integration of multiple layers of biological data (e.g., genomics, transcriptomics, proteomics, metabolomics) to obtain a comprehensive view of biological systems.
Normalization	Adjustment of data values to remove bias caused by technical variation, enabling meaningful comparison between samples
Overfitting	When a model performs well on training data but poorly on unseen data, usually due to excessive complexity
Oversampling/Undersampling	Oversampling generate synthetic minority class samples, while undersampling reduces the majority class
PCA (Principal Component Analysis)	A linear dimensionality reduction method that transforms correlated features into a set of uncorrelated components
Proteomics	Large-scale study of proteins, their expression levels, structures, and functions within a biological sample
RNN (Recurrent Neural Network)	Neural network architecture designed to process sequential data such as time series or ordered omics measurements
Underfitting	When a model is too simple to capture underlying patterns in data, resulting in poor performance on both training and test sets

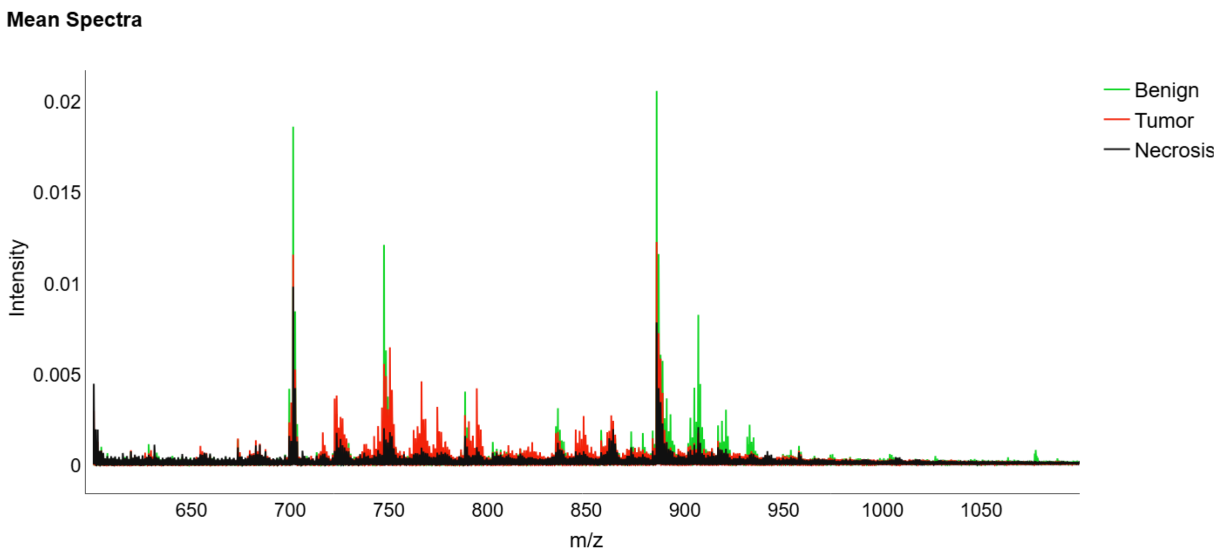

Supplementary Figure 2. Display of a mean mass spectrum acquired using a conventional mass spectrometer (from Waters), showing intensity profiles across *m/z* for three tissue types: benign, tumor, and necrotic.

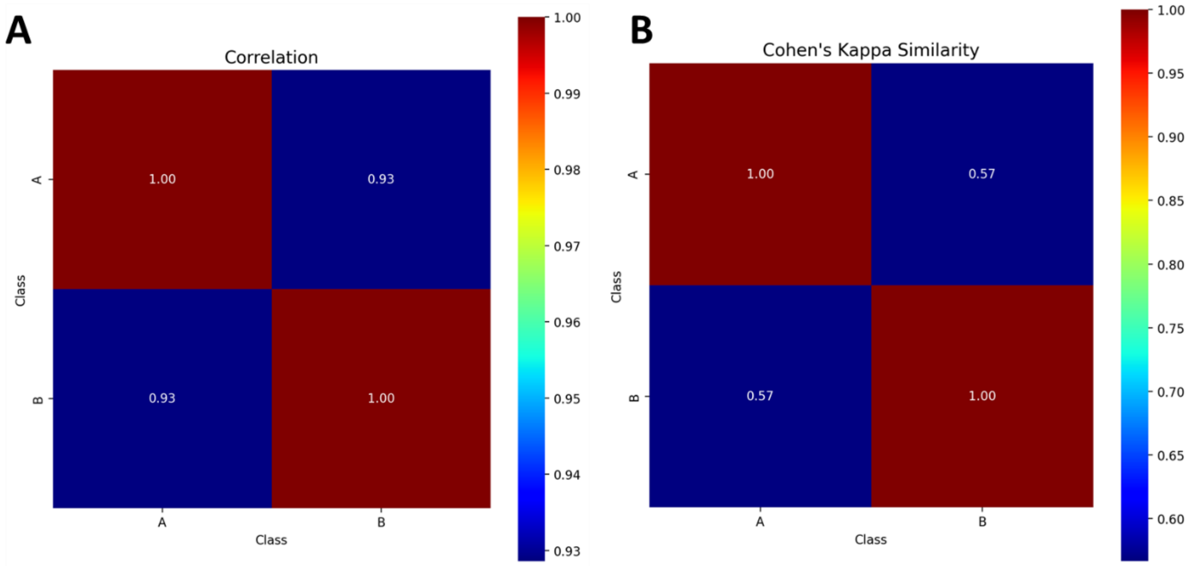

Supplementary Figure 3. Results using the correlation and similarity module of Profiler. **A)** Spearman correlation matrix and **B)** Cohen's kappa Similarity matrix with 6 discretization levels.

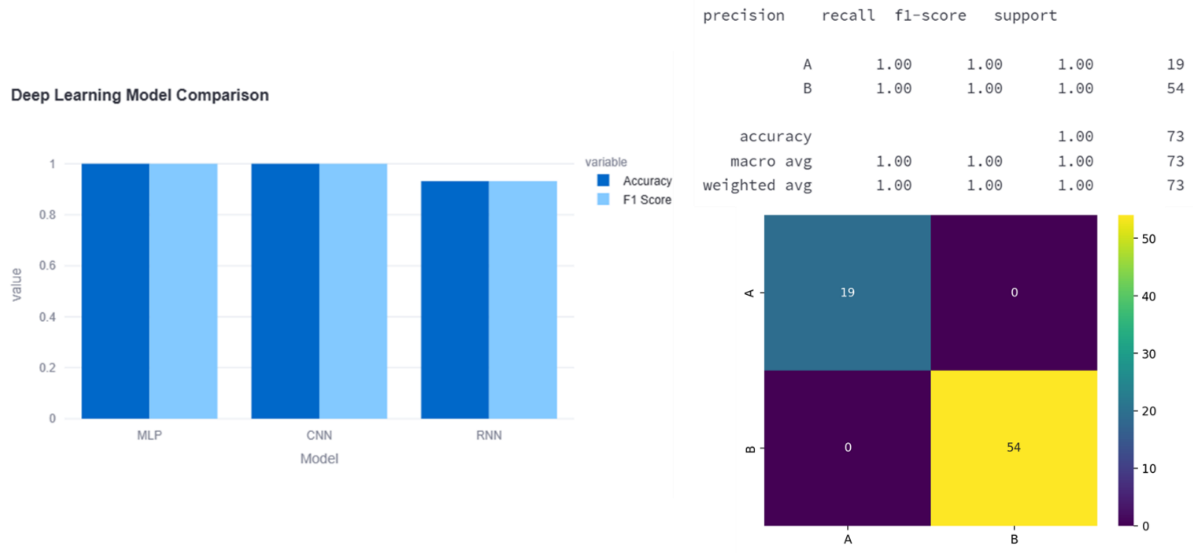

Supplementary Figure 4. Deep Learning results where three model accuracies are compared, classification metrics and the confusion matrix for the best-performing model is detailed.

Supplementary Table 2. Short glossary of key technical terms used in this article.

**Model Type**	**Architectures**	**Parameters**	**Accuracy**	**F1-score**	**Relative Training Time**
Machine Learning	RandomForest, LightGBM, SVC, Logistic Regression…	Default (Scikit-learn)	0.55–0.95	0.54–0.93	Fast (~1.25 min) (for all models)
Deep Learning	MLP, CNN, RNN (LSTM)	Epochs=10, Batch=32, LR=0.001 (Adam)	0.86–0.91	0.86–0.91	Moderate (~ 6 min)
↳ MLP	2 dense layers (128–64), Dropout=0.3	Same as above	0.91	0.90	Fast (~10 sec)
↳ CNN	Conv1D (32–64), kernel=3, Dropout=0.3	Same as above	0.91	0.91	Fast (~20sec)
↳ RNN	2 LSTM layers (64–32), Dropout=0.3	Same as above	0.86	0.86	Slow (~5 min)

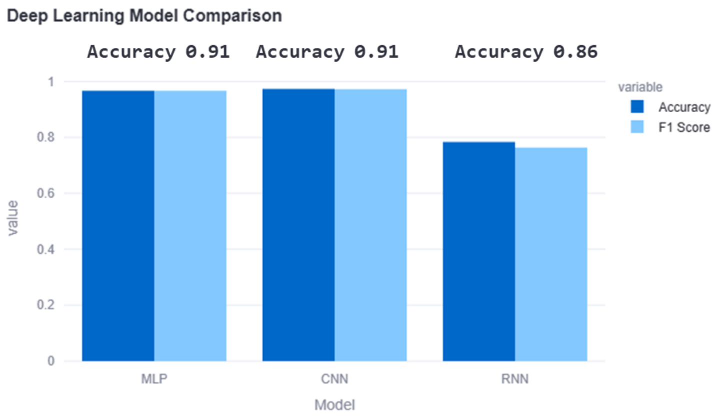

Supplementary Figure 5. Deep Learning results where three model accuracies are compared with their accuracies detailed. These results were obtained after KNN imputation of the matrix, using a 70% presence threshold.

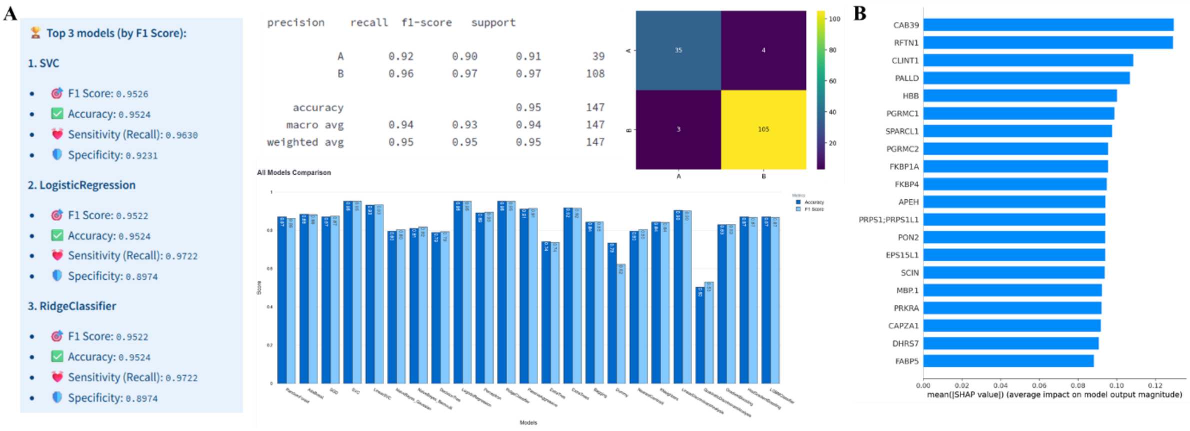

Supplementary Figure 6. **A**) Model accuracies are compared, top 3 models, classification metrics, confusion matrix and learning curve for the best-performing model is detailed. Here for the dataset preprocessed using KNN imputation with a 70% presence threshold. **B**) Average SHAP values for the 20 proteins that most strongly influence the model's predictions.

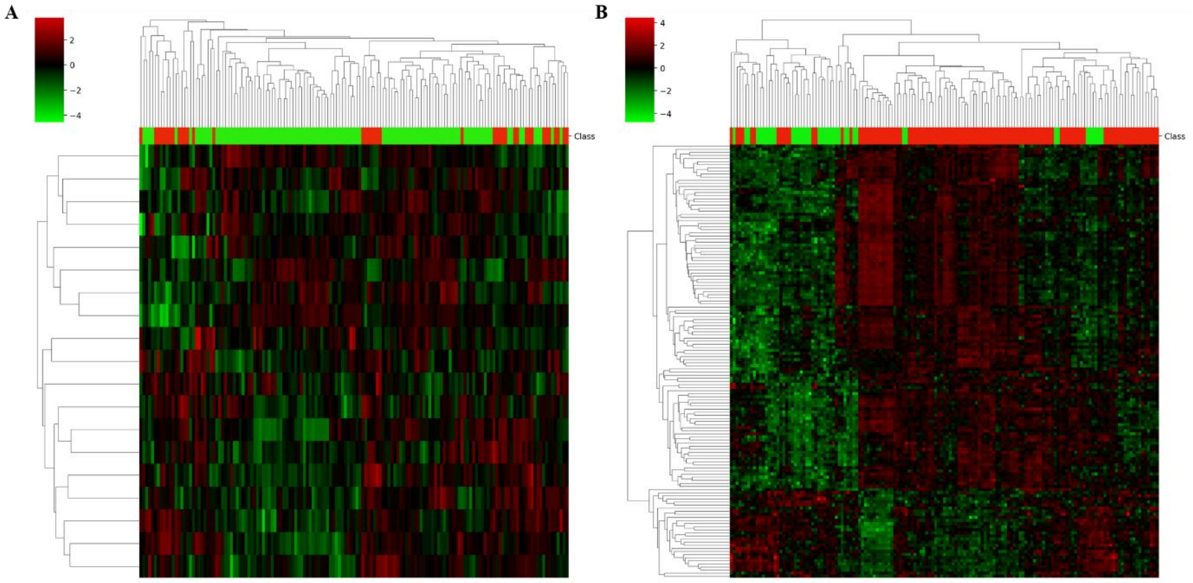

Supplementary Figure 7. Heatmap made with a p-value of 0.01 based on **A)** AI explainaibility biomarkers or **B)** volcano plot biomarkers

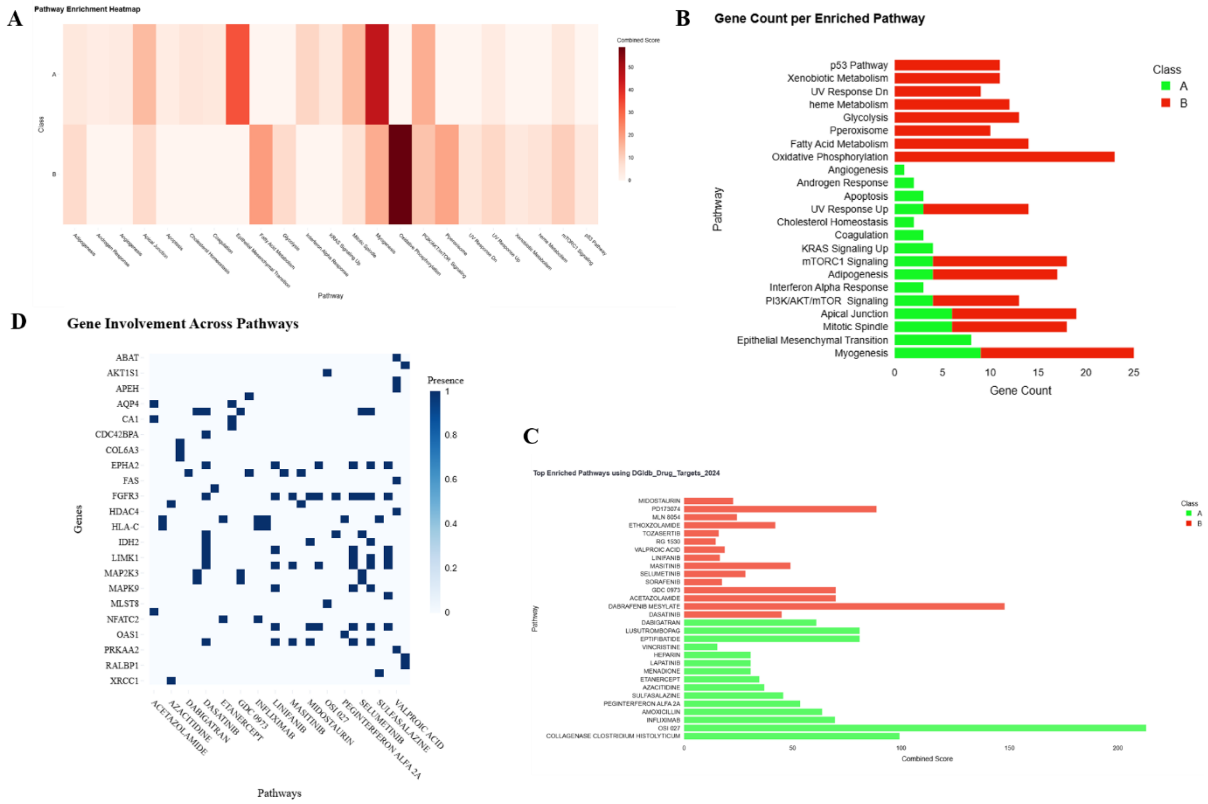

Supplementary Figure 8. Panels with **A**) enriched pathways heatmap according to combined score, **B**) enriched drugs according to gene count, **C**) enriched drugs bar plot according to combined score and **D**) gene involvement across drugs depicted as an heatmap

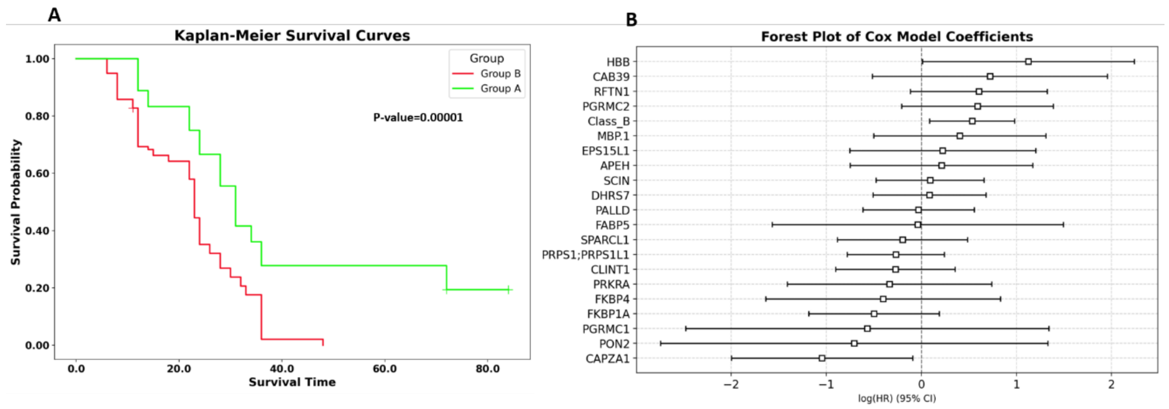

Supplementary Figure 9. **A)** Survival curves (Kaplan Meier analysis) of all 50 patients according to the two groups. **B)** Forest plot of Cox model coefficients made with top 20 contributing biomarkers to the survival outcome.

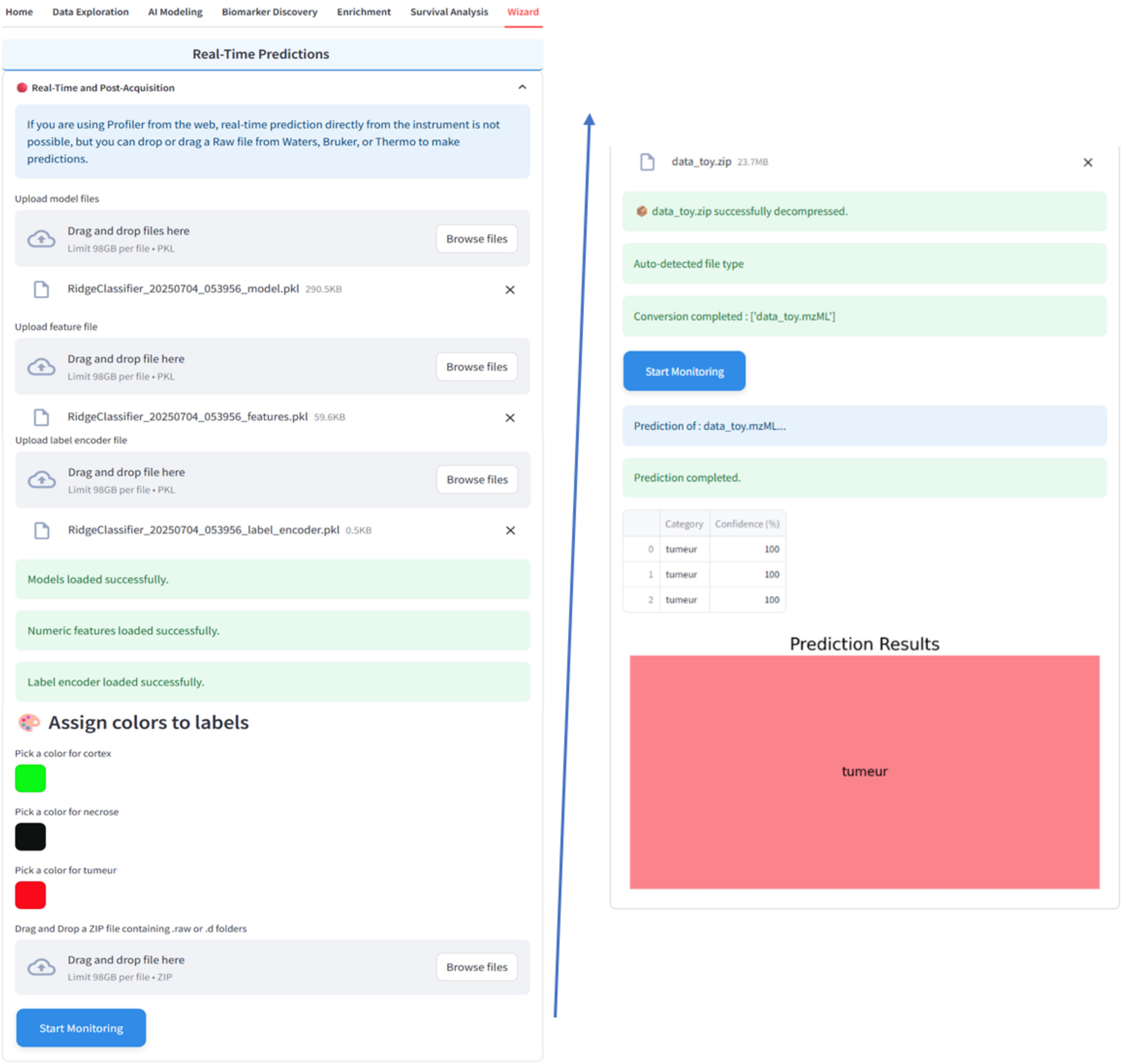

Supplementary Figure 10. Blind results using the Wizard module of Profiler, especially the real-time and post-acquisition one.

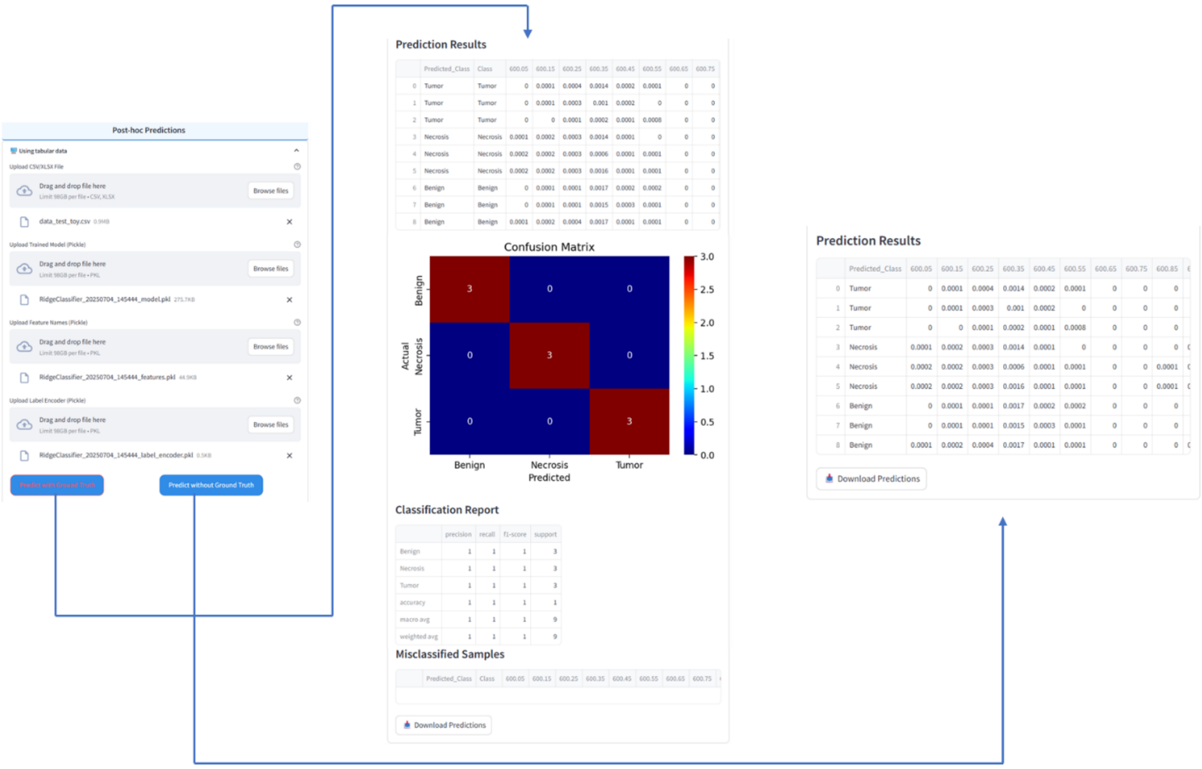

Supplementary Figure 11. Blind results using the Wizard module of Profiler, especially post-hoc one.

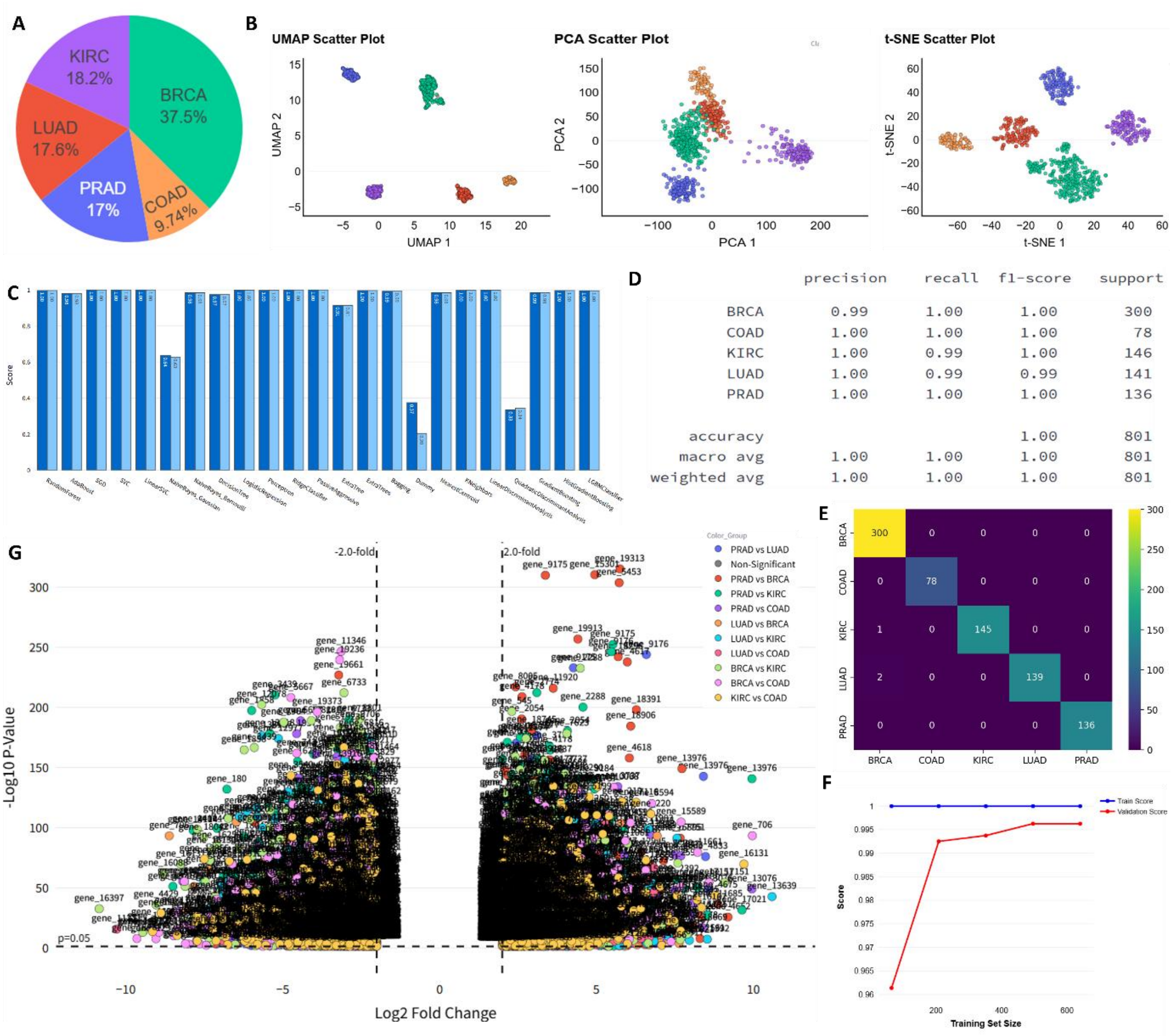

Supplementary Figure 12. Results obtained with Profiler on a transcriptomic dataset comparing 5 cancer types for a pan-cancer analysis (BRCA: Breast Invasive Carcinoma, KIRC: Kidney Renal Clear Cell Carcinoma, COAD: Colon Adenocarcinoma, LUAD: Lung Adenocarcinoma and PRAD: Prostate Adenocarcinoma). **A**) Proportion of samples for each cancer type, **B**) Unsupervised learning using 3 dimensionality reduction algorithms, **C-D-E-F**) Model accuracies are compared, classification metrics, confusion matrix and learning curve for the best-performing model is detailed and **G**) Volcano plot with a 1-fold change threshold and p-value of 0.05, showing up- and down-regulated proteins depending on the cancer type.

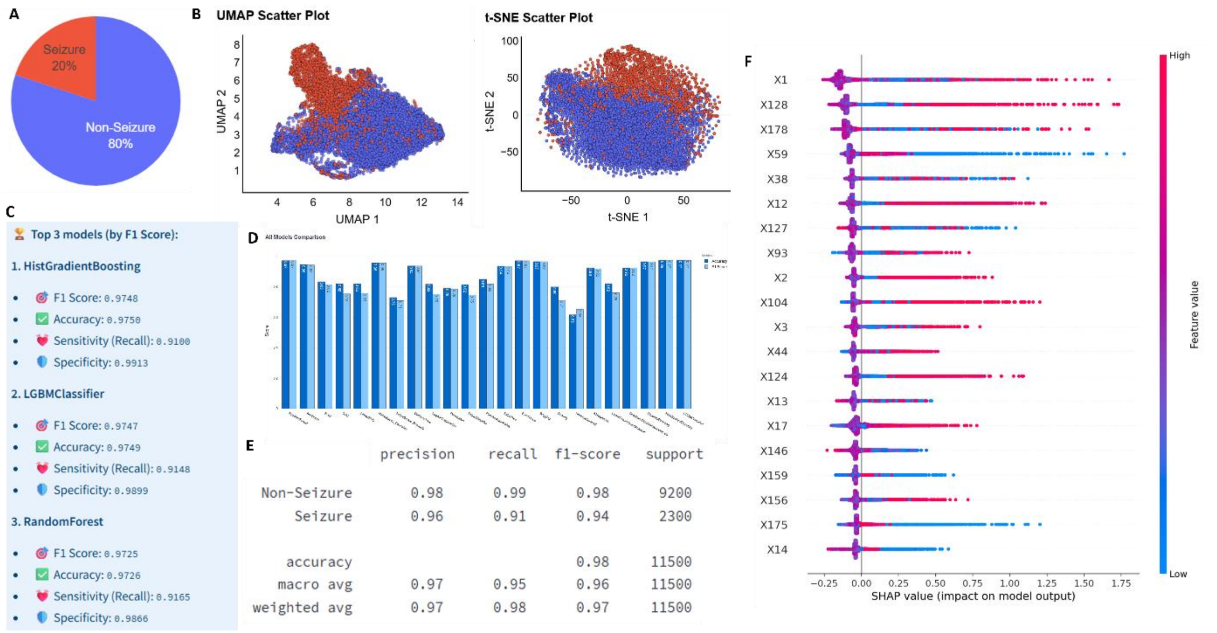

Supplementary Figure 13. Results obtained with Profiler on a EEG dataset comparing EEGs of patients who have or have not had a seizure. **A**) Proportion of samples for each class, **B**) Unsupervised learning using two dimensionality reduction algorithms, **C-D-E**) Model accuracies are compared, top 3 models and classification metrics for the best-performing model is detailed and **F**) SHAP beeswarm plot of the top 20 proteins contributing to the best ML model.

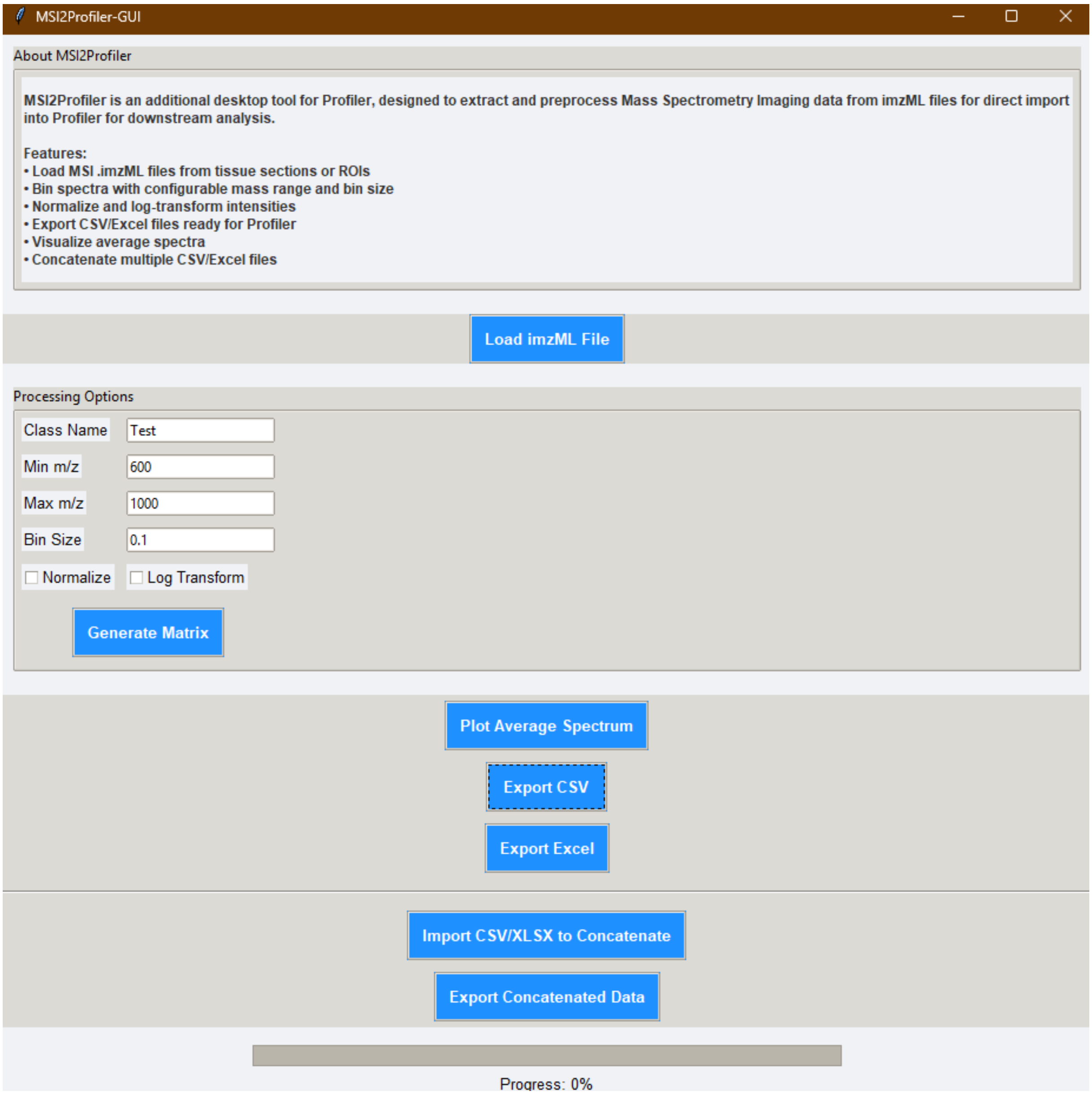

Supplementary Figure 14. Screenshot of the MSI2Profiler tool.
